# γ-Alumina Nanoparticle Catalyzed Efficient Synthesis of Highly Substituted Imidazoles

**DOI:** 10.3390/molecules201019221

**Published:** 2015-10-21

**Authors:** Bandapalli Palakshi Reddy, Vijayaparthasarathi Vijayakumar, Mariadhas Valan Arasu, Naif Abdullah Al-Dhabi

**Affiliations:** 1 Center for Organic and Medicinal Chemistry, VIT University, Vellore 632014, Tamil Nadu, India; E-Mail: palakshireddy@gmail.com; 2 Department of Botany and Microbiology, Addiriyah Chair for Environmental Studies, College of Science, King Saud University, P. O. Box 2455, Riyadh 11451, Saudi Arabia; E-Mails: mvalanarasu@gmail.com (M.V.A.); naifaldhabi2014@gmail.com (N.A.A.)

**Keywords:** benzil, arylaldehydes, γ-Alumina NPs, one pot synthesis, 1,2,4,5-tetraaryl imidazoles

## Abstract

γ-Alumina nano particle catalyzed multi component reaction of benzil, arylaldehyde and aryl amines afforded the highly substituted 1,2,4,5-tetraaryl imidazoles with good to excellent yield in less reaction time under the sonication as well as the conventional methods. Convenient operational simplicity, mild conditions and the reusability of catalyst were the other advantages of this developed protocol.

## 1. Introduction

**Figure 1:**
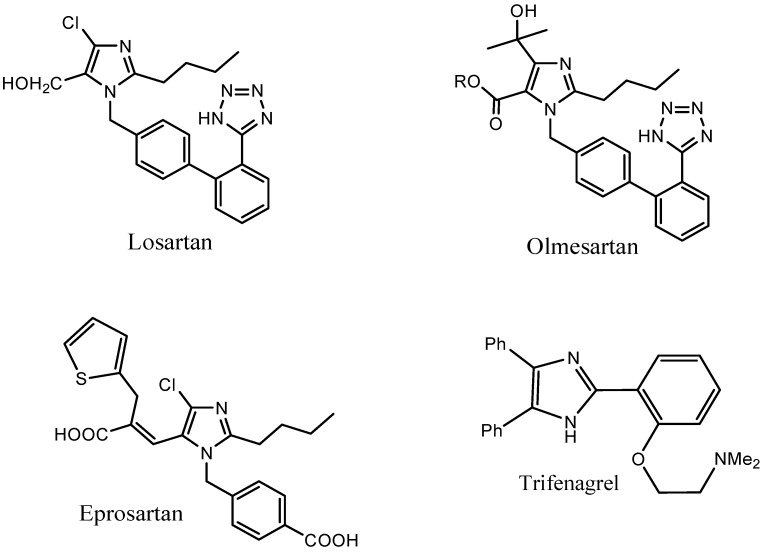
Some of the imidazole based drugs.

The imidazole ring system was reported [1] as an active component of several drugs such as Losartan, Olmesartan, Eprosartan and Trifenagrel (Figure 1), and many biologically important compounds like histidine, histamine and biotin. The potency and pertinence of imidazole pharmacophore was largely due to its hydrogen-bond donor acceptor nature as well as its high affinity towards the metals existing in the protein active sites (e.g., Fe, Zn, Mg). The imidazole derivatives were reported to function as inhibitors of p38 MAP kinase, B-Raf kinase [2], transforming growth factor b1 (TGF-b1) type 1 activin receptor-like kinase (ALK5) [3], cyclooxygenase-2 (COX-2) [4] and were also reported to be involved in the biosynthesis of interleukin-1 (IL-1) [5,6]. Appropriately substituted imidazoles were used as glucagon receptors [7], CB1 cannabinoid receptor antagonists [8] and modulators of P-glycoprotein (P-gp) mediated multidrug resistance (MDR) [9]. The imidazole core was also reported to exhibit anti-allergic [10], anti-inflammatory [11], analgesic, antifungal, antimycotic, antibiotic, anti-ulcerative, antibacterial and antitumor activity [12].

A number of methods have been developed for the synthesis of 1,2,4,5-tetrasubstituted imidazoles. The catalysts, such as silica gel or Zeolite HY [13], silica gel/NaHSO_4_ [14], I_2_ [15], K_5_CoW_12_O_40_•3H_2_O [16], heteropoly acids [17], HClO_4_-SiO_2_ [18], InCl_3_·3H_2_O [19], ZrCl_4_ [20], BF_3_•SiO_2_ [21], DABCO [22], PEG-400 [23] and silica-bonded propylpiperazine *N*-sulfamic acid (SBPPSA) [24], were also served for this purpose, all these methods suffered by several disadvantages like the usage of expensive moisture sensitive catalysts, hazardous organic solvents, laborious workup, longer reaction duration, larger volume of catalyst. Hence the development of a mild, simple, more efficient and green procedure for the synthesis of 1,2,4,5-tetrasubstituted imidazoles was highly desirable.

In recent years, nano catalysts have gained prominence due to their efficiency and selectivity. The easy work up and reusability were the added advantages associated with the usage of this type of catalysts. Alumina is one of the inert biomaterial used in implants due to its biocompatible nature [25,26,27,28,29,30]. It exists in many metastable forms (γ, δ, θ, κ, ε, η, χ) and in particular γ-Al_2_O_3_ has significant applications as a catalyst [31]. γ-Al_2_O_3_ is *iso*-structural with γ-Fe_2_O_3_ and perhaps the most important nano material used as a support for metal catalysts; in view of its inherent properties like environmental compatibility, greater selectivity, moisture-insensitivity and operational simplicity, we intend to explore the catalytic behavior of γ-Al_2_O_3_ NPs in the synthesis of imidazoles. We anticipated that the Lewis acid behavior and the smaller particle size of Al_2_O_3_ NPs (with large surface area) may efficiently catalyze the chemical reaction. Hence we attempted the γ-Al_2_O_3_ NPs catalyzed synthesis of tetraaryl imidazoles which is hitherto unreported.

## 2. Results and Discussion

To the aqueous the solution of Al(NO_3_)_3_•9H_2_O (1.72 g dissolved in 460 mL of distilled water) ammonia solution (30 mL) was added in drop wise using peristaltic pump under stirring with a propeller at 500 rpm for 30 min. The resulted turbid solution was warmed at 90 °C (using a temperature controlled water bath) till all the aluminum hydroxide was precipitated. The precipitate was collected by centrifugation and washed with distilled water followed by ethanol and then calcinated at 80 °C for four hours. The overall reaction for the synthesis of Al_2_O_3_ NPs from Al(NO_3_)_3_ can be depicted as,

Al(NO_3_)_3_ + 3NH_4_OH → Al(OH)_3_ ↓ + 3NH_4_NO_3_ (1)

2Al(OH)_3_ → 2AlOOH(boehmite) + 2H_2_O (2)

2AlOOH(boehmite) → γ-Al_2_O_3_+ H_2_O (3)

**Figure 2:**
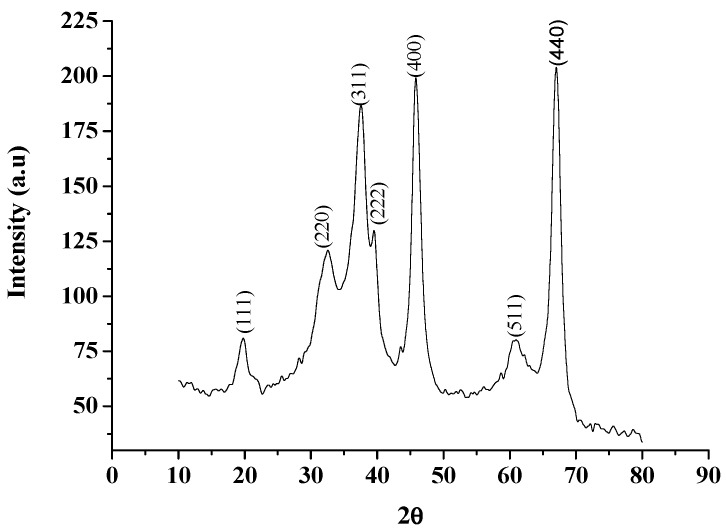
XRD pattern of γ-Al_2_O_3_ NPs.

**Figure 3:**
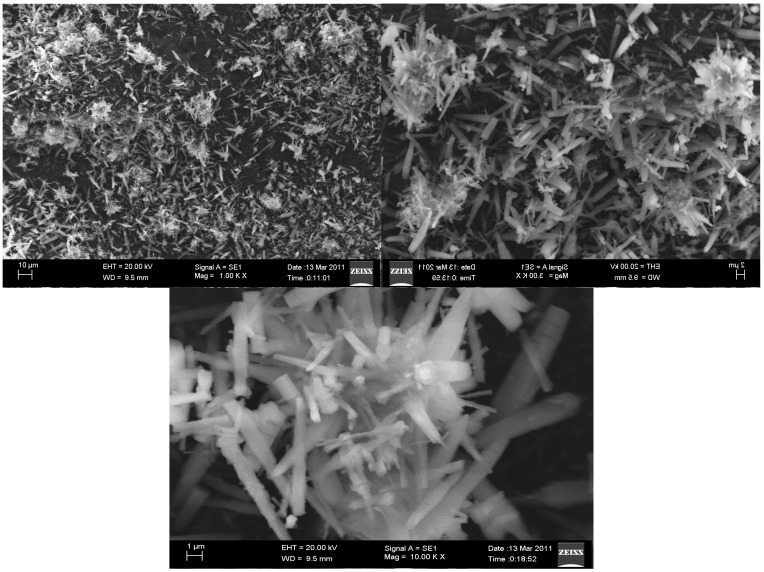
SEM micrographs of synthesized γ-Al_2_O_3_ NPs at various magnifications.

**Scheme 1:**
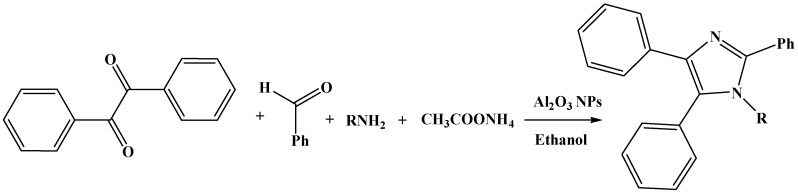
Tetraaryl substituted imidazoles using multi-component reaction.

The synthesized Al_2_O_3_ NPs were characterized using Power X-ray Diffractometer with Cu Kα radiation (λ = 1.54 Å) over a 2θ range of 10°–90°. The XRD pattern exhibited seven distinct diffraction peaks at 19.79, 32.54, 37.53, 39.01, 45.81, 60.92 and 66.98 which could be assigned to (1 1 1), (2 2 0), (3 1 1), (2 2 2), (4 0 0), (5 1 1) and (4 4 0) of cubic nano γ-Al_2_O_3_ respectively and found to be in agreement with the database of JCPDS No. 00-010-0425 (Joint Committee on Powder Diffraction Standards) (Figure 2). The images of γ-Al_2_O_3_ NPs were observed using SEM (Carl Zeiss oxford instrument, (Oxford, UK) at various magnifications (Figure 3). The micrograph at lower magnification revealed the formation of well dispersed rod shaped γ-Al_2_O_3_ NPs. After confirming the formation of γ-Al_2_O_3_ NPs it was subjected as a catalyst in the synthesis of tetraaryl imidazoles via multi component reaction (Scheme 1) of benzil (1 mmol), arylaldehyde (1 mmol), ammonium acetate (2.0 mmol), and aniline (1 mmol) in ethanol. The observed yield and reaction duration of γ-Al_2_O_3_ NPs catalyzed reaction conferred that the γ-Al_2_O_3_ was an effective and efficient catalyst as anticipated. The observed efficiency may be attributed to Lewis acid behavior of γ-Al_2_O_3_ NPs and its smaller particle size (larger surface area). For optimization the reaction of benzil (1 mmol), 4-hydroxybenzaldehyde (1 mmol), ammonium acetate (2.0 mmol) and 4-methylaniline (1 mmol) in ethanol was chosen as a representative reaction for the synthesis of 4-(4,5-diphenyl-1-(4-methylphenyl)-1*H*-imidazol-2-yl)phenol. Catalytic efficiency was investigated under sonication and conventional heating methods. In conventional method the reaction mixture was refluxed for 240 min in the absence of catalyst, in this the observed yield was 33% of imidazole **8**, but the same reaction under similar conditions in the presence of 5 mol % of Al_2_O_3_ NPs could yield 82% of imidazole **8** in 60 min. The increase in mol % of Al_2_O_3_ NPs from 5 mol % to 10 mol % not only decreased the reaction time from 60 min to 40 min but also increased the yield of imidazole **8** from 82% to 93% (Table 1). Further increase in concentration of γ-Al_2_O_3_ NPs has no effect on the yield and time of the reaction.

**Table 1:** Catalytic activity evaluation at 78 °C for synthesis of tetraaryl imidazole **8** in ethanol.

In Conventional Method				Under Ultrasonication			
Entry	Al_2_O_3_ (mol %)	Time (min)	Yield (%) ^b^	Entry	Al_2_O_3_ (mol %)	Time (min)	Yield (%) ^b^
**1**	20	40	93	**1**	20	25	95
**2**	15	40	93	**2**	15	25	94
**3**	10	40	93	**3**	10	25	94
**4**	05	60	82	**4**	05	45	80
**5**	00	240	33	**5**	00	120	35

^b^ Isolated yield.

**Figure 4:**
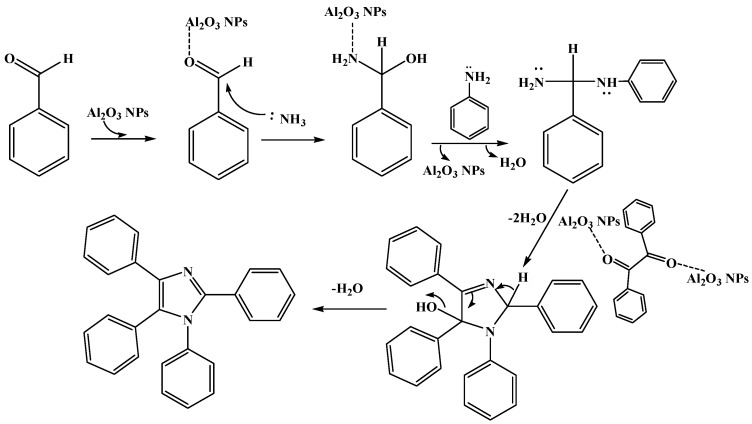
Plausible mechanism for synthesis of tetraaryl imidazoles.

**Figure 5:**
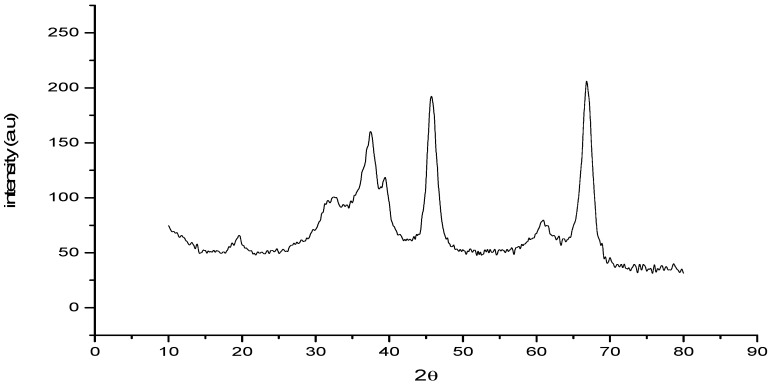
XRD pattern of recovered γ-Al_2_O_3_ NPs after four runs.

**Figure 6:**
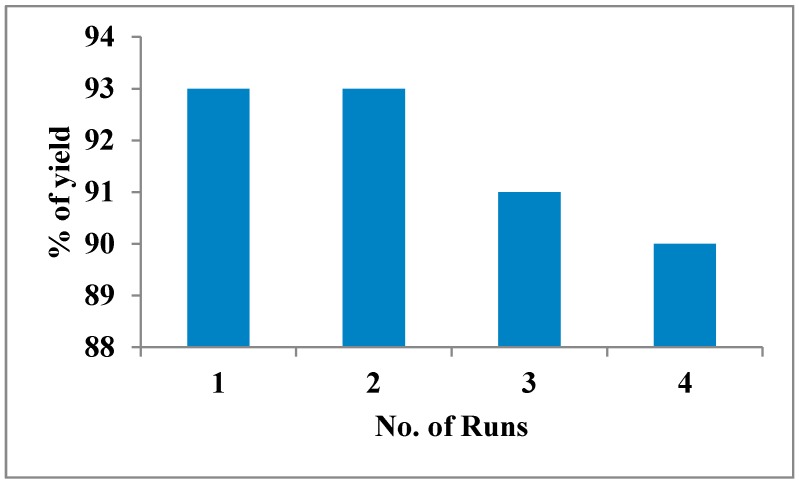
Reusability of catalyst.

The effect of temperature on the reaction was investigated by carrying out the representative reaction at different temperatures (RT (25 °C), 50 °C, 80 °C and 100 °C) in solvents like acetonitrile and ethanol separately with 10 mol % of the catalyst and found that the yield was not affected with the increase of temperature (Table 2). To investigate the effects of media, the reaction was carried out in polar and non-polar solvents at RT using 10 mol % γ-Al_2_O_3_ NPs the catalyst at 80 °C (maximum of 78 °C temperature was maintained when ethanol was used as solvent). The polar solvents were found to be much better than non-polar solvents. Though acetonitrile, dichloromethane or ethanol were found to be good solvents (Table 3), ethanol was opted as a suitable solvent since it is relatively environmental benign and it required only the aqueous work up. The same model reaction was carried under sonication (to compare the general conventional process) and found that the yields were significantly increased under sonication (Table 1); this may be due to the dispersion phenomenon. The required concentration of catalyst under the sonication was investigated by changing its concentration in the synthesis of imidazole **8** and found that 10 mol % was sufficient to afford imidazole with 94% yield in 35 min (Table 1). The excellent yield in lesser time (compared to the conventional process) may be due to the availability of large surface area of catalyst and the sonication assisted dispersion of NPs. The reaction of benzil with various arylaldehydes bearing electron withdrawing groups (such as nitro, halide, *etc.*) or electron releasing groups (methyl, hydroxyl; mono, di, or tri methoxy groups, *etc.*), benzyl amine, aniline derivatives and ammonium acetate were also successfully carried out in the presence of γ-Al_2_O_3_ NPs. After optimizing the conditions a series of tetraaryl imidazoles from **1**–**22** were synthesized successfully (Table 4). Good to excellent yield of desired products was observed (without the formation of 2,4,5-trisubstituted imidazoles as side products, which were normally observed under the influence of the strong acids [19]). Plausible mechanism of the synthesis of tetraaryl imidazoles given in (Figure 4). The protocol described for the synthesis of tetraaryl imidazoles possesses its scope in the context of ease, generality and the simplicity. Waste generation and side products were largely avoided and hence the products were obtained with high yield and purity. In this experiment, after the completion of reaction, the reusability of the catalyst was assessed by washing the filtered catalyst (Figure 5) thoroughly with ethanol and distilled water followed by activation of the catalyst at 250 °C for 2 h (Figure 6). The separated catalyst was reused efficiently for four cycles with consistent activity (yields were 93%, 93%, 91% and 90%). All these tetraaryl imidazoles **1**–**22** were synthesized using the same methodology and characterized through IR, ^1^H-NMR, ^13^C-NMR and Mass spectral data and were available as supplementary data.

**Table 2:** Temperature evaluation and effect of solvent in the synthesis of tetraaryl imidazole **8**.

Temperature Evaluation ^a^			
Entry	Temperature (°C)	Time (min)	Yield (%) ^c^
1	25	90	88
2	50	60	90
3	78	40	93

^a^ in ethanol and 10 mol % catalyst; ^c^ Isolated yields.

**Table 3:** Effect of solvent on the yield of tetraaryl imidazole **8** at 10 mol % catalyst.

Effect of Solvent ^b^		
Entry	Solvent	Yield (%) ^c^
1	Ethanol	93
2	Methanol	88
3	Dichloromethane	86
4	Acetonitrile	88

^b^ at reflux temp, time 40 min; ^c^ Isolated yields.

**Table 4:** Synthesis of tetraaryl imidazoles (**1**–**22**) ^a^.

Entry	R	Ph	Reaction Time (min)		Yield (%) ^b^		mp (°C)
			Conventional	US	Conventional	US	
1	-CH_2_Ph	Ph	40	25	92	95	161–163
2	-CH_2_Ph	4-ClPh	40	25	92	94	165–167
3	-CH_2_Ph	4-OC_2_H_5_Ph	50	30	92	94	155–157
4	-CH_2_Ph	3,5-(OCH_3_)_2_Ph	50	35	92	93	180–182
5	-CH_2_Ph	3-Cl Ph	40	25	92	93	144–146
6	4-CH_3_Ph	4-OH-3-OC_2_H_5_Ph	45	25	90	94	180–182
7	4-CH_3_Ph	4-C_2_H_5_Ph	55	30	90	93	212–214
8	4-CH_3_Ph	4-OHPh	40	25	93	94	>275
9	4-CH_3_Ph	3,5-(OCH_3_)_2_Ph	45	30	91	94	140–142
10	4-CH_3_Ph	3,4,5-(OCH_3_)_3_Ph	55	35	93	93	102–104
11	4-CH_3_Ph	2-Thienyl	40	25	89	92	200–201
12	4-OCH_3_Ph	3,4,5-(OCH_3_)_3_Ph	60	45	91	92	123–125
13	4-ClPh	4-C_2_H_5_Ph	55	35	92	93	181–182
14	4-ClPh	3,4,5-(OCH_3_)_3_Ph	60	40	91	93	123–125
15	4-ClPh	4-CNPh	60	45	87	89	112–114
16	4-ClPh	AllyloxyPh	60	50	91	91	98–100
17	4-ClPh	4-BrPh	50	35	93	91	80–82
18	4-IPh	2,4-(Cl)_2_Ph	45	25	89	92	109–111
19	4-IPh	4-OH-3-OCH_3_Ph	50	30	93	94	96–98
20	4-CH_3_Ph	3-OHPh	50	25	93	93	260–162
21	4-ClPh	3-OHPh	45	30	94	92	85–87
22	4-ClPh	4-OH-3-OC_2_H_5_Ph	40	30	94	94	169–170

^a^ Reaction conditions: aldehyde (1 mmol), aniline (1 mmol) and γ-Al_2_O_3_ NPs (10 mol %), ethanol (10 mL), ammonium acetate (2.0 mmol); ^b^ Isolated and unoptimized yields.

In summary, the reaction of arylaldehyde, aryl amine and ammonium acetate with benzil in the presence of γ-Al_2_O_3_ NPs as efficient and effective catalyst provided a simple one-pot entry into the synthesis of biologically active highly substituted imidazoles. The simplicity, efficiency, generality, high yield, eco-friendly procedure, reusability of the catalyst was the promising points of the described methodology.

## 3. Experimental Section

### 3.1. Chemicals and Apparatus

Solvents and reagents were commercially sourced and used without further purification. Melting points were recorded on Elchem Microprocessor (Chennai, India) based DT apparatus in open capillary tubes and are uncorrected. IR spectra recorded on Avatar-330 FTIR spectrophotometer (Thermo Nicolet, Madison, WI, USA) using KBr pellets, and only noteworthy absorption levels (reciprocal centimeters) has been listed. Sonication was carried out by using E-Chrom ultrasonic horn (10F-8, No. 20, Minchuan W. Road, Taipei 104, Taiwan), 22 kHz frequency. The NMR spectra were recorded on Bruker (Bruker Corporation, Billerica, MA, USA) 400 & 500 MHz spectrometers using TMS as internal standard (chemical shifts δ in ppm). CDCl_3_ and DMSO-*d*_6_ are used as NMR solvents. Mass spectra were recorded on an HRMS MicromasszQ (San Diego, CA, USA) spectrometer. TLC was performed on preparative plates of silica gel (s.d.fine). Visualization was made with an iodine chamber.

### 3.2. Preparation of Al_2_O_3_ NPs

In a typical preparation, Al(NO_3_)_3_•9H_2_O (1.72 g) was dissolved in 460 mL distilled water and 30 mL of ammonia solution added drop wise using peristaltic pump under stirring using a propeller at 500 rpm for 30 min. The resulting turbid solution was warmed for two hours using a temperature controlled water bath at 90 °C till all aluminum hydroxide settled. The resulting precipitate was harvested by centrifugation, washed with distilled water followed by ethanol. The precipitate was then calcined at 800 °C for four hours.

*General procedure for the synthesis of 1,2,4,5-tetraaryl imidazoles (***1**–**22***) under the conventional heating:* An aldehyde (1 mmol), aniline (1 mmol) and γ Al_2_O_3_ NPs (10 mol %) in ethanol (10 mL), were added, stirred for 10 min. To this ammonium acetate (2.0 mmol) followed by 1,2-diketone (1 mmol) were added, then the reaction mixture was heated at 80 °C until completion of the reaction. Completion of the reaction was monitored by TLC. The reaction mixture was cooled to RT and catalyst was filtered, the solvent was removed by rotary evaporator. The crude product was dissolved in ethyl acetate and water (3 × 10 mL:10 mL). The organic layer was separated and dried over anhydrous Na_2_SO_4_ and then the solvent was distilled under reduced pressure to get crude product. The crude product was purified by column chromatography to afford the corresponding imidazoles in good to excellent yield.

*General procedure for the synthesis of 1,2,4,5-tetraaryl imidazoles (***1**–**22***) under the ultrasonication:* To ethanol (10 mL), aldehyde (1 mmol), aniline (1 mmol) and γ Al_2_O_3_ NPs (10 mol %) in ethanol (10 mL) were added and stirred for 10 min. To this ammonium acetate (2.0 mmol) followed by 1,2-diketone (1 mmol) were added, then the reaction mixture was kept under sonicationup to the completion of the reaction (Table 4). Completion of the reaction was monitored by TLC. The reaction mixture was cooled to RT and catalyst was filtered, the solvent was removed by rotary evaporator. The crude product was dissolved in ethyl acetate and water (3 × 10 mL:10 mL). The organic layer was separated and dried over anhydrous Na_2_SO_4_ and then the solvent was distilled under reduced pressure to get crude product. The crude product was purified by column chromatography to afford the corresponding imidazoles in good to excellent yield. The identity as well as purity of the product was confirmed by ^1^H-, ^13^C-NMR, and mass spectra.

## 4. Spectral Data

*1-Benzyl-2,4,5-triphenyl-1H-imidazole* (**1**): Yield: 95%. m.p.: 161–163 °C; IR (KBr, cm^−1^): 2956, 1613, 1560, 1416. ^1^H-NMR (400 MHz, CDCl_3_) δ_H_: 5.11 (s, 2H), 6.80 (d, *J =* 7.4 Hz, 3H), 7.10 (t, *J =* 7.4 Hz, 1H), 7.2–7.4 (m, 10H), 7.52 (d, *J =* 7.6 Hz, 2H), 7.56 (t, *J =* 7.6 Hz, 3H), 7.18–7.24 (m, 8H), 7.28–7.34 (m, 3H) 7.63 (d, *J =* 6.8 Hz, 2H). ^13^C-NMR (100 MHz, CDCl_3_) δ_C_: 31.03, 48.41, 76.84, 77.16, 77.47, 115.85, 116.06, 116.15, 116.38, 124.60, 124.63, 126.00, 126.11, 126.46, 126.61, 126.88, 127.61, 128.18, 128.23, 128.67, 128.69, 128.80, 128.88, 128.97, 129.14, 130.01, 130.25, 130.33, 130.62, 130.85, 131.14, 133.03, 133.08, 133.11, 134.36, 135.02, 137.35, 138.39, 146.73, 146.75, 161.55, 164.00, 194.70, 207.12. HRMS (*m*/*z*): Calcd. for C_28_H_22_N_2_: 386.1783. Found: 386.1788 (M^+^).

*1-Benzyl-2-(4-chlorophenyl)-4,5-diphenyl-1H-imidazole* (**2**): Yield: 94%. m.p.: 165–167 °C; IR (KBr, cm^−1^): 2986, 1618, 1563, 1417, 802. ^1^H-NMR (500 MHz, DMSO-*d*_6_) δ_H_: 5.12 (s, 2H), 6.83 (d, *J =* 7.4 Hz, 2H), 7.19 (t, *J =* 7.4 Hz, 1H), 7.32 (t, *J =* 7.4 Hz, 1H), 7.20–7.40 (m, 6H), 7.52 (d, *J =* 7.6 Hz, 3H), 7.56 (t, *J =* 7.6 Hz, 3H), 7.69 (d, *J =* 6.8 Hz, 3H). ^13^C-NMR (125 MHz, DMSO) δ_C_: 48.30, 125.88, 126.03, 126.36, 126.50, 126.80, 127.35, 127.52, 128.07, 128.13, 128.23, 128.57, 128.58, 128.61, 128.72, 128.76, 128.78, 128.83, 128.86, 128.90, 129.03, 129.09, 129.90, 130.07, 130.43, 130.98, 131.05, 131.10, 134.30, 134.48, 135.00, 137.34, 137.56, 138.11, 138.32, 138.50, 146.86. HRMS (*m*/*z*): Calcd. for C_28_H_21_ClN_2_: 420.1393. Found: 420.1387 (M^+^).

*1-Benzyl-2-(4-ethoxyphenyl)-4,5-diphenyl-1H-imidazole* (**3**): Yield: 94%. m.p.: 155–157 °C; IR (KBr, cm^−1^): 2965, 1629, 1598, 1423, 1134. ^1^H-NMR (400 MHz, CDCl_3_) δ_H_: 1.41 (t, *J =* 7.2 Hz, 3H), 4.10 (q, *J =* 7.2 Hz, 2H), 5.10 (s, 2H), 6.79 (d, *J =* 7.4 Hz, 2H), 6.89 (d, *J =* 7.4 Hz, 2H), 7.23–7.56 (m, 10H), 7.54 (d, *J =* 7.4 Hz, 2H), 7.97 (d, *J =* 7.2 Hz, 4H). ^13^C-NMR (100 MHz, CDCl_3_) δ_C_: 14.91, 29.50, 48.38, 63.64, 114.70, 123.33, 126.17, 126.42, 126.94, 127.45, 128.19, 128.67, 128.71, 128.89, 129.17, 129.23, 129.87, 130.06, 130.57, 131.24, 131.30, 134.67, 137.82, 137.94, 148.22, 159.65. HRMS (*m*/*z*): Calcd. for C_3_0H_26_N_2_O: 430.2045. Found: 430.2033 (M^+^).

*1-Benzyl-2-(3,5-dimethoxyphenyl)-4,5-diphenyl-1H-imidazole* (**4**): Yield: 93%. m.p.: 180–182 °C; IR (KBr, cm^−1^): 2945, 1685, 1531, 1492, 1176. ^1^H-NMR (500 MHz, DMSO-*d*_6_) δ_H_: 3.67 (s, 3H), 3.87 (s, 3H), 5.08 (s, 2H), 6.87 (d, *J =* 6.8 Hz, 3H), 7.10 (d, *J =* 7.2 Hz, 1H), 7.14 (s, 1H), 7.18–7.24 (m, 8H), 7.28–7.34 (m, 3H), 7.56 (d, *J =* 6.8 Hz, 2H). ^13^C-NMR (125 MHz, DMSO) δ_C_: 48.31, 55.71, 55.99, 111.05, 112.29, 121.69, 123.56, 125.99, 126.44, 126.91, 127.44, 128.17, 128.73, 128.77, 128.92, 130.02, 131.13, 134.57, 137.89, 138.01, 148.06, 148.84, 149.67. HRMS (*m*/*z*): Calcd. for C_30_H_26_N_2_O_2_: 446.1994. Found: 446.1980 (M^+^).

*1-Benzyl-2-(3-chlorophenyl)-4,5-diphenyl-1H-imidazole* (**5**): Yield: 93%. m.p.: 144–146°C; IR (KBr, cm^−1^): 2980, 1610, 1521, 1410, 1122, 790. ^1^H-NMR (400 MHz, CDCl_3_) δ_H_: 5.12 (s, 2H), 6.82 (s, 2H), 7.19–7.29 (m, 8H), 7.34–7.41 (m, 8H), 7.61 (t, *J =* 7.4 Hz, 1H), 7.67 (d, *J =* 7.4 Hz, 1H). ^13^C-NMR (100 MHz, CDCl_3_) δ_C_: 48.30, 125.87, 126.02, 126.40, 126.53, 126.81, 127.38, 127.54, 128.11, 128.16, 128.26, 128.59, 128.62, 128.64, 128.73, 128.80, 128.82, 128.85, 128.89, 128.95, 129.08, 129.41, 129.90, 130.11, 130.26, 130.48, 130.76, 130.93, 131.03, 131.08, 134.31, 134.47, 134.98, 137.32, 137.53, 138.06, 138.28, 138.48, 146.85, 148.09, 148.45. HRMS (*m*/*z*): Calcd. for C_28_H_21_ClN_2_: 420.1393. Found: 420.1399 (M^+^).

*4-(4,5-Diphenyl-1-(4-methylphenyl)-1H-imidazol-2-yl)-2-ethoxyphenol* (**6**): Yield: 94%. m.p.: 180–182 °C; IR (KBr, cm^−1^): 2956, 1613, 1560, 1416, 1139. ^1^H-NMR (500 MHz, DMSO-*d*_6_) δ_H_: 1.32 (t, 3H, *J =* 7.6 Hz), 2.30 (s, 3H), 3.90 (q, 2H, *J =* 7.6 Hz), 6.84 (d, 1H, *J =* 7.6 Hz), 6.75 (s, 1H), 6.91 (d, 2H, *J =* 7.6 Hz), 7.00–7.06 (m, 3H), 7.10–7.25 (m, 6H), 7.58 (d, 2H, *J =* 7.6 Hz), 7.97 (d, 2H, *J =* 7.6 Hz). ^13^C-NMR (125 MHz, DMSO) δ_C_: 14.89, 21.23, 31.06, 64.42, 112.70, 114.13, 122.46, 122.79, 126.60, 127.53, 127.93, 128.23, 128.35, 128.41, 129.16, 129.79, 130.03, 130.65, 130.94, 131.26, 133.10, 134.66, 134.84, 135.03, 137.92, 138.20, 145.41, 146.13, 147.14, 194.72, 207.16. HRMS (*m*/*z*): Calcd. for C_30_H_26_N_2_O_2_: 446.1994. Found: 446.1981 (M^+^).

*2-(4-Ethylphenyl)-4,5-diphenyl-1-(4-methylphenyl)-1H-imidazole* (**7**): Yield: 93%. m.p.: 212–214 °C; IR (KBr, cm^−1^): 2967, 1694, 1523, 1461, 1245. ^1^H-NMR (400 MHz, CDCl_3_) δ_H_: 1.19 (s, 3H), 2.31 (s, 3H), 2.61 (m, 2H), 6.90 (d, *J =* 7 Hz, 1H), 7.00 (d, *J =* 8.2 Hz, 2H), 7.15–7.42 (m, 6H), 7.52 (d, *J =* 7.4 Hz, 2H), 7.58 (d, *J =* 7.4 Hz, 2H), 7.65 (t, *J =* 8 Hz, 2H), 8.00 (d, *J =* 8 Hz, 2H). ^13^C-NMR (100 MHz, CDCl_3_) δ_C_: 15.36, 21.31, 28.70, 31.06, 125.47, 126.59, 127.53, 127.70, 127.92, 127.98, 128.11, 128.23, 128.27, 128.40, 128.45, 128.68, 128.95, 129.16, 129.78, 130.04, 130.84, 130.99, 131.27, 133.11, 134.72, 135.04, 138.17, 144.43, 147.24. HRMS (*m*/*z*): Calcd. for C_30_H_26_N_2_: 414.2096. Found: 414.2090 (M^+^).

*4-(4,5-Diphenyl-1-(4-methylphenyl)-1H-imidazol-2-yl)phenol* (**8**): Yield: 94%. m.p.: >280 °C; IR (KBr, cm^−1^): 2956, 1619, 1562, 1414, 1287. ^1^H-NMR (400 MHz, CDCl_3_) δ_H_: 2.26 (s, 3H), 6.65 (d, *J =* 7.4 Hz, 2H), 7.08–7.24 (m, 7H), 7.32 (d, *J =* 7.4 Hz, 3H), 7.49 (d, *J =* 7.4 Hz, 2H), 7.68 (t, *J =* 7.2 Hz, 2H), 7.81 (t, *J =* 7 Hz, 3H), 7.95 (d, *J =* 5.6 Hz, 2H). ^13^C-NMR (100 MHz, CDCl_3_) δ_C_: 21.09, 115.40, 121.86, 126.69, 126.77, 128.53, 128.71, 128.87, 128.93, 129.97, 130.01, 130.05, 130.23, 131.08, 131.20, 131.60, 132.74, 134.79, 135.12, 135.99, 136.83, 138.33, 158.01. HRMS (*m*/*z*): Calcd. for C_28_H_22_N_2_O: 402.1732. Found: 402.1720 (M^+^).

*2-(3,5-Dimethoxyphenyl)-4,5-diphenyl-1-(4-methylphenyl)-1H-imidazole* (**9**): Yield: 94%. m.p.: 140–142 °C; IR (KBr, cm^−1^): 2923, 1609, 1567, 1495, 1165. ^1^H-NMR (400 MHz, CDCl_3_) δ_H_: 2.20 (s, 3H), 3.60 (s, 6H), 5.55 (d, 1H), 6.71 (d, *J =* 7.2 Hz, 2H), 6.95 (d, *J =* 7.2 Hz, 2H), 7.22 (d, *J =* 7.4 Hz, 2H), 7.40–7.60 (m, 6H), 7.97 (d, *J =* 7.2 Hz, 4H). ^13^C-NMR (100 MHz, CDCl_3_) δ_C_: 29.83, 56.14, 106.16, 126.70, 127.56, 128.02, 128.28, 128.46, 128.49, 129.17, 129.83, 130.06, 130.86, 131.28, 133.14, 135.03, 138.02, 138.32, 146.63, 147.04. HRMS (*m*/*z*): Calcd. for C_30_H_26_N_2_O_2_: 446.1994. Found: 446.1980 (M^+^).

*4,5-Diphenyl-1-(4-methylphenyl)-2-(3,4,5-trimethoxyphenyl)-1H-imidazole* (**10**): Yield: 93%. m.p.: 102–104 °C; IR (KBr, cm^−1^): 2934, 1693, 1567, 1436, 1173. ^1^H-NMR (400 MHz, CDCl_3_) δ_H_: 2.30 (s, 3H), 3.61 (s, 6H), 3.81 (s, 3H), 6.70 (s, 3H), 6.90 (d, *J =* 8 Hz, 3H), 7.10 (d, *J* = 8 Hz, 2H), 7.30–7.50 (m, 5H), 7.50 (t, *J* = 6.8 Hz, 2H), 7.60 (d, *J* = 6.8 Hz, 2H), 7.70 (d, *J* = 6.8 Hz, 1H), 8.00 (d, *J* = 7.4 Hz, 2H). ^13^C-NMR (100 MHz, CDCl_3_) δ_C_: 21.16, 55.88, 60.96, 106.28, 125.91, 126.72, 127.51, 128.04, 128.26, 128.39, 128.44, 129.13, 129.83, 130.01, 130.72, 131.01, 131.21, 133.08, 134.51, 134.87, 135.01, 138.08, 138.11, 138.37, 146.74, 152.77. HRMS (*m*/*z*): Calcd. for C_31_H_28_N_2_O_3_: 476.2100. Found: 476.2109 (M^+^).

*4,5-Diphenyl-2-(thiophen-2-yl)-1-(4-methylphenyl)-1H-imidazole* (**11**): Yield: 92%. m.p.: 200–201 °C; IR (KBr, cm^−1^): 2959, 1643, 1562, 1414, 1165. ^1^H-NMR (400 MHz, CDCl_3_) δ_H_: 2.30 (s, 3H), 6.9 (d, *J* = 7.6 Hz, 2H), 7.1 (d, *J*= 7.6 Hz, 2H), 7.3–7.5 (m, 6H), 7.51 (t, *J =* 6.8 Hz, 2H), 7.57 (d, *J =* 7.2 Hz, 2H), 7.65 (t, *J =* 6.8 Hz, 1H), 7.82 (d, *J* = 7.2 Hz, 1H), 7.97 (d, *J* = 7.2 Hz, 4H), 8.47 (d, *J* = 7.4 Hz, 1H), 8.59 (s, 1H). ^13^C-NMR (100 MHz, CDCl_3_) δ_C_: 14.24, 126.89, 123.12, 126.99, 127.41, 128.10, 128.23, 128.33, 128.51, 129.13, 130.01, 130.14, 130.41, 131.16, 131.75, 133.07, 134.02, 134.29, 135.01, 136.07, 138.87, 138.92, 144.14, 149.00, 149.51. HRMS (*m*/*z*): Calcd. for C_27_H_22_N_2_S: 406.1501. Found: 406.1501 (M^+^).

*1-(4-Methoxyphenyl)-4,5-diphenyl-2-(3,4,5-trimethoxyphenyl)-1H-imidazole* (**12**): Yield: 92%. m.p.: 123–125 °C; IR (KBr, cm^−1^): 2909, 1667, 1549, 1492, 1174. ^1^H-NMR (500 MHz, DMSO-*d*_6_) δ_H_: 3.68 (s, 6H), 3.78 (s, 3H), 3.84 (s, 3H), 6.71 (s, 2H), 6.82 (d, *J =* 6 Hz, 3H), 7.00 (d, *J =* 7.2 Hz, 2H), 7.19–7.32 (m, 7H), 7.50 (t, *J =* 7.2 Hz, 2H), 7.60 (d, *J =* 7.8 Hz, 2H), 7.68 (t, *J =* 7.2 Hz, 1H), 7.98 (d, *J =* 6 Hz, 3H) ^13^C-NMR (125 MHz, DMSO) δ_C_: 55.48, 55.88, 60.86, 106.23, 114.29, 125.90, 126.61, 127.39, 127.95, 128.16, 128.37, 129.03, 129.58, 129.91, 130.15, 130.69, 131.08, 131.13, 133.02, 134.46, 134.89, 137.94, 138.10, 146.74, 152.73, 159.25. HRMS (*m*/*z*): Calcd. for C_31_H_28_N_2_O_4_: 492.2049. Found: 492.2040 (M^+^).

*1-(4-Chlorophenyl)-2-(4-ethylphenyl)-4,5-diphenyl-1H-imidazole* (**13**): Yield: 93%. m.p.: 181–182 °C; IR (KBr, cm^−1^): 2996, 1687, 1564, 1436, 802. ^1^H-NMR (400 MHz, CDCl_3_) δ_H_: 1.41 (t, *J =* 6.8 Hz, 3H), 4.03 (d, *J =* 6.8 Hz, 2H), 6.78 (d, *J =* 8.2 Hz, 2H), 6.94 (d, *J =* 8.2 Hz, 2H), 7.19 (d, *J =* 8 Hz, 2H), 7.31 (m, 3H), 7.52 (d, *J =* 8 Hz, 2H), 7.55 (m, 3H), 7.64 (d, *J =* 7.6 Hz, 2H), 7.97 (d, *J =* 7.6 Hz, 2H). ^13^C-NMR (100 MHz, CDCl_3_) δ_C_: 63.57, 114.34, 121.15, 122.73, 126.78, 127.52, 128.24, 128.30, 128.65, 129.17, 129.44, 129.78, 130.06, 130.35, 130.53, 130.64, 131.26, 133.14, 134.14, 134.45, 135.04, 135.93, 138.37, 147.16, 159.30.HRMS (*m*/*z*): Calcd. for C_29_H_23_ClN_2_: 434.1550. Found: 434.1558 (M^+^).

*1-(4-Chlorophenyl)-4,5-diphenyl-2-(3,4,5-trimethoxyphenyl)-1H-imidazole* (**14**): Yield: 93%. m.p.: 123–125 °C; IR (KBr, cm^−1^): 2990, 1667, 1513, 1454, 782. ^1^H-NMR (400 MHz, CDCl_3_) δ_H_: 3.64 (s, 3H), 3.75 (s, 6H), 3.87 (s, 3H), 6.68 (s, 2H), 6.80 (d, *J =* 8.2 Hz, 3H), 7.00 (d, *J =* 8.2 Hz, 2H), 7.01–7.03 (m, 4H), 7.50 (t, *J =* 7.4 Hz, 2H), 7.60 (d, *J =* 7.4 Hz, 2H), 7.96 (d, *J =* 8 Hz, 1H), 7.98 (d, *J =* 8 Hz, 2H). ^13^C-NMR (100 MHz, CDCl_3_) δ_C_: 55.61, 56.00, 61.00, 106.30, 114.40, 126.01, 126.74, 127.51, 128.07, 128.29, 128.50, 129.16, 129.70, 130.04, 130.24, 130.78, 131.19, 131.25, 133.11, 134.55, 135.04, 138.04, 138.16, 146.87, 152.84, 159.35. HRMS (*m*/*z*): Calcd. for C_30_H_25_ClN_2_O_3_: 496.1554. Found: 496.1540 (M^+^).

*4-(1-(4-Chlorophenyl)-4,5-diphenyl-1H-imidazol-2-yl)benzonitrile* (**15**): Yield: 89%. m.p.: 112–114 °C; IR (KBr, cm^−1^): 2947, 1698, 1512, 1498, 805.^1^H-NMR (400 MHz, CDCl_3_) δ_H_: 6.98 (d, 1H, *J =* 7.2 Hz) 7.11 (d, 1H, *J =* 7.2 Hz), 7.21–7.31 (m, 4H), 7.51–7.56 (m, 6H), 7.66 (t, 2H, *J =* 7.4 Hz), 7.97 (d, 4H, *J =* 7.4 Hz). ^13^C-NMR (100 MHz, CDCl_3_) δ_C_: 111.89, 118.68, 121.14, 127.24, 127.41, 128.44, 128.72, 128.82, 129.11, 129.14, 129.17, 129.56, 129.90, 130.05, 131.14, 132.12, 133.11, 133.82, 134.56, 135.05, 135.27, 136.62, 139.42, 144.77, 168.43, 194.75, 207.20. HRMS (*m*/*z*): Calcd. for C_28_H_18_ClN_3_: 431.1189. Found: 431.1180 (M^+^).

*2-(4-(Allyloxy)phenyl)-1-(4-chlorophenyl)-4,5-diphenyl-1H-imidazole* (**16**): Yield: 91%. m.p.: 98–100 °C; IR (KBr, cm^−1^): 2956, 1613, 1560, 1416, 1187. ^1^H-NMR (400 MHz, CDCl_3_) δ_H_: 4.51 (s, 2H), 5.27 (d, *J =* 7.6 Hz, 1H), 5.38 (d, *J =* 7.6 Hz, 1H), 6.03 (t, *J =* 8 Hz, 1H), 6.80 (d, *J =* 8 Hz, 2H), 6.95 (d, *J =* 8 Hz, 2H), 7.18 (d, *J =* 8 Hz, 2H), 7.32 (d, *J =* 7.2 Hz, 2H), 7.4–7.6 (m, 6H), 7.97 (d, *J =* 7.2 Hz, 4H). ^13^C-NMR (100 MHz, CDCl_3_) δ_C_: 68.88, 114.61, 117.97, 121.15, 123.05, 126.80, 127.51, 128.26, 128.30, 128.65, 129.08, 129.17, 129.45, 129.77, 130.05, 130.39, 130.51, 130.60, 131.25, 133.07, 133.13, 134.18, 134.41, 135.04, 135.89, 138.39, 147.05, 158.93. HRMS (*m*/*z*): Calcd. for C_30_H_23_ClN_2_O: 462.1499. Found: 462.1490 (M^+^).

*2-(4-Bromophenyl)-1-(4-chlorophenyl)-4,5-diphenyl-1H-imidazole* (**17**): Yield: 91%. m.p.: 80–82 °C; IR (KBr, cm^−1^): 2956, 1665, 1560, 1489, 783. ^1^H-NMR (400 MHz, CDCl_3_) δ_H_: 6.98 (d, *J =* 7.4 Hz, 2H), 7.13 (d, *J =* 7.4 Hz, 2H), 7.41–7.55 (m, 6H), 7.68 (t, *J =* 7.2 Hz, 4H), 7.99 (d, *J =* 7.2 Hz, 4H). ^13^C-NMR (100 MHz, CDCl_3_) δ_C_: 24.58, 121.21, 123.08, 127.02, 127.48, 128.37, 128.48, 128.72, 129.06, 129.17, 129.64, 130.03, 130.50, 131.18, 131.62, 133.12, 134.11, 134.61, 135.04, 135.51, 136.69, 138.85, 168.53. HRMS (*m*/*z*): Calcd. for C_27_H_18_BrClN_2_: 484.0342. Found: 484.0349 (M^+^).

*2-(2,4-Dichlorophenyl)-1-(4-iodophenyl)-4,5-diphenyl-1H-imidazole* (**18**): Yield: 92%. m.p.: 109–111 °C; IR (KBr, cm^−1^): 2945, 1609, 1554, 1417, 786. ^1^H-NMR (400 MHz, CDCl_3_) δ_H_: 6.66 (d, *J =* 7.2 Hz, 1H) 7.14 (d, *J =* 7.2 Hz, 1H), 7.17–7.33 (m, 3H), 7.46–7.58 (m, 7H), 7.66 (t, *J =* 7.4 Hz, 2H), 7.97 (d, *J =* 7.4 Hz, 3H). ^13^C-NMR (100 MHz, CDCl_3_) δ_C_: 93.86, 121.75, 127.05, 127.24, 127.54, 128.35, 128.49, 128.82, 128.99, 129.16, 129.47, 129.72, 129.92, 130.03, 131.00, 133.07, 133.66, 133.95, 135.05, 135.54, 135.86, 136.33, 137.93, 138.07, 138.59, 143.92, 168.57. HRMS (*m*/*z*): Calcd. for C_27_H_17_Cl_2_IN_2_: 565.9813. Found 565.9819 (M^+^).

## 5. Reusability of the Catalyst

In the experiment, after the reaction was completed, the γ-Al_2_O_3_ NPs catalyst was isolated from the reaction mixture by filtration in the work-up stage. The reusability of the catalyst was assessed by washing thoroughly by ethanol and distilled water followed by activating the catalyst at 250 °C for 2 h. The separated catalyst was reused efficiently for four cycles with consistent activity and yields are 93%, 93%, 91% and 90% (Figure 6).

## 6. Conclusions

In conclusion, the reaction of aldehyde, aryl amine and ammonium acetate with benzyl in ethanol in the presence of γ Al_2_O_3_ NPs as an efficient and effective catalyst provides a simple one-pot entry into the synthesis of highly substituted imidazole derivatives. The promising points of the present methodology were efficiency, generality, high yield, eco-friendliness, reusability of the catalyst and simplicity process for the preparation of 1,2,4,5-tetrasubstituted imidazoles.

## Supplementary Materials

Supplementary materials can be accessed at: http://www.mdpi.com/1420-3049/20/10/19221/s1.

## References

[1] Grimmett M.R., Katritzky A.R., Rees C.W., Katritzky A.R., Rees C.W. (1984). Comprehensive Heterocyclic Chemistry.

[2] Takle A.K., Brown M.J.B., Davies S., Dean D.K., Francis G., Gaiba A., Hird A.W., King F.W., Lovell P.J., Naylor A. (2006). The identification of potent and selective imidazole-based inhibitors of B-Raf kinase. Bioorg. Med. Chem. Lett..

[3] Khanna I.K., Weier R.M., Yu Y., Xu X.D., Koszyk F.J., Collins P.W., Koboldt C.M., Veenhuizen A.W., Perkins W.E., Casler J.J. (1997). 1,2-Diarylpyrroles as Potent and Selective Inhibitors of Cyclooxygenase-2. J. Med. Chem..

[4] Lange J.H.M., Van-Stuivenberg H.H., Coolen K.K.A.C., Adolfs T.J.P., McCreary A.C., Keizer H.G., Wals H.C., Veerman W., Borst A.J.M., de Loof P.C. (2005). Bioisosteric replacements of the pyrazole moiety of rimonabant, synthesis, biological properties, and molecular modeling investigations of thiazoles, triazoles, and imidazoles as potent and selective CB1 cannabinoid receptor antagonists. J. Med. Chem..

[5] Gallagher T.F., Fier-Thompson S.M., Garigipati R.S., Sorenson M.E., Smietana J.M., Lee D., Bender P.E., Lee J.C., Laydon J.T., Griswold D.E. (1995). 2,4,5-triarylimidazole inhibitors of IL-1 biosynthesis. Bioorg. Med. Chem. Lett..

[6] Laufer S.A., Zimmermann W., Ruff K.J. (2004). Tetrasubstituted imidazole inhibitors of cytokine release: Probing substituents in the N-1 position. J. Med. Chem..

[7] De Laszlo S.E., Hacker C., Li B., Kim D., MacCoss M., Mantlo N., Pivnichny J.V., Colwell L., Koch G.E., Cascieri M.A. (1999). Potent, orally absorbed glucagon receptor antagonists. Bioorg. Med. Chem. Lett..

[8] Eyers P.A., Craxton M., Morrice N., Cohen P., Goedert M. (1998). Conversion of SB 203580-insensitive MAP kinase family members to drug-sensitive forms by a single amino-acid substitution. Chem. Biol..

[9] Newman M.J., Rodarte J.C., Benbatoul K.D., Romano S.J., Zhang C., Krane S., Moran E.J., Uyeda R.T., Dixon R., Guns E.S. (2000). Discovery and characterization of OC144-093, a novel inhibitor of P-glycoprotein-mediated multidrug resistance. Cancer Res..

[10] Misono M. (2001). Unique acid catalysis of heteropoly compounds (heteropolyoxometalates) in the solid state. Chem. Commun..

[11] Black J.W., Durant G.J., Emmett J.C., Ganellin C.R. (1974). Sulphurmethylene isosterism in the development of metiamide, a new histamine H2-receptor antagonist. Nature.

[12] Ucucu U., Karaburun N.G., Iskdag I. (2001). Synthesis and analgesic activity of some 1-benzyl-2-substituted-4, 5-diphenyl-1*H*-imidazole derivatives. IL Farm..

[13] Balalaei S., Arabanian A. (2000). One-pot synthesis of tetrasubstituted imidazoles catalyzed by zeolite HY and silica gel under microwave irradiation. Green Chem..

[14] Karimi A.R., Alimohammadi Z., Azizian J., Mohammadi A.A., Mohmmadizade M.R. (2006). Solvent-free synthesis of tetrasubstituted imidazoles on silica gel/NaHSO_4_ support. Catal. Commun..

[15] Kidwai M., Mothsra P., Bansal V., Somvanshi R.K., Ethayathulla A.S., Dey S., Singh T.P. (2007). One-pot synthesis of highly substituted imidazoles using molecular iodine: A versatile catalyst. J. Mol. Catal. A Chem. 2.

[16] Nagarapu L., Apuri S., Kantevari S. (2007). Potassium dodecatugstocobaltate trihydrate (K_5_CoW_12_O_40_•3H_2_O): A mild and efficient reusable catalyst for the one-pot synthesis of 1,2,4,5-tetrasubstituted imidazoles under conventional heating and microwave irradiation. J. Mol. Catal. A Chem..

[17] Heravi M.M., Derikvand F., Bamoharram F.F. (2007). Highly efficient, four-component one-pot synthesis of tetrasubstituted imidazoles using Keggin-type heteropolyacids as green and reusable catalysts. J. Mol. Catal. A Chem..

[18] Kantevari S., Vuppalapati S.V.N., Biradar D.O., Nagarapu L. (2007). Synthesis of 1,2,4,5-tetrasubstituted imidazoles using silica-bonded propylpiperazine *N*-sulfamic acid as a recyclable solid acid catalyst. J. Mol. Catal. A Chem..

[19] Sharma S.D., Hazarika P., Konwar D. (2008). An efficient and one-pot synthesis of 2,4,5-trisubstituted and 1,2,4,5-tetrasubstituted imidazoles catalyzed by InCl_3_•3H_2_O. Tetrahedron Lett..

[20] Sharma G.V., Jyothi Y., Lakshmi P.S. (2006). Efficient room-temperature synthesis of tri- and tetrasubstituted imidazoles catalyzed by ZrCl_4_. Synth. Commun..

[21] Sadeghi B., Mirjalili B.F., Hashemi M.M. (2008). BF_3_•SiO_2_ an efficient reagent system for the one-pot synthesis of 1,2,4,5-tetrasubstituted imidazoles. Tetrahedron Lett..

[22] Murthy S.N., Madhav B., Nageswar Y.V.D. (2010). DABCO as a mild and efficient catalytic system for the synthesis of highly substituted imidazoles via multi-component condensation strategy. Tetrahedron Lett..

[23] Wang X.C., Gong H.P., Quan Z.J., Li L., Ye H.L. (2009). PEG-400 as an efficient reaction medium for the synthesis of 2,4,5-triaryl-1*H*-imidazoles and 1,2,4,5-tetraaryl-1*H*-imidazoles. Chin. Chem. Lett..

[24] Niknam K., Deris A., Naeimi F., Majleci F. (2011). Synthesis of 1,2,4,5-tetrasubstituted imidazoles using silica-bonded propylpiperazine Nsulfamic acid as a recyclable solid acid catalyst. Tetrahedron Lett..

[25] Zielínski P.A., Schulz R., Kaliaguine S., van Neste A. (1993). Structural transformations of alumina by high energy ball milling. J. Mater. Res..

[26] Hanawa T., Kaga M., Itoh Y., Echizenya T., Oguchi H., Ota M. (1992). Cytotoxicities of oxides, phosphates and sulphides of metals. Biomaterials.

[27] Dey S., Bakthavatchalu V., Tseng M.T., Wu P., Florence R.L., Grulke E.A., Yokel R.A., Sanjit K.D., Yang H.S., Chen Y. (2008). Interactions between SIRT1 and AP-1 reveal a mechanistic insight into the growth promoting properties of alumina (Al_2_O_3_) nanoparticles in mouse skin epithelial cells. Carcinogenesis.

[28] Oesterling E., Chopra N., Gavalas V., Arzuaga X., Lim E.J., Sultana R., Butterfield L., Bachas D.A., Hennig B. (2008). Alumina nanoparticles induce expression of endothelial cell adhesion molecules. Toxicol. Lett..

[29] Hussain S.M., Hess K.L., Gearhart J.M., Geiss K.T., Schlager J.J. (2005). *In vitro* toxicity of nanoparticles in BRL 3A rat liver cells. Toxicol. In Vitro.

[30] Cordingley R., Kohan L., Ben-Nissan B., Pezzotti G. (2003). Alumia and Zirconia bioceramics in orthopaedic applications. J. Aust. Ceram. Soc..

[31] Guevara-Lara A., Bacaud R., Vrinat M. (2007). Highly active NiMo/TiO_2_-Al_2_O_3_ catalysts: Influence of the preparation and the activation conditions on the catalytic activity. Appl. Catal. A Gen..

