# Supplementary material for: γ-Alumina Nanoparticle Catalyzed Efficient Synthesis of Highly Substituted Imidazoles
Source: Molecules. 2015 Oct 21;20(10):19221–35. doi: 10.3390/molecules201019221 (PMC6332066; doi:10.3390/molecules201019221)
Supplement: Supplementary file 1 [file molecules-20-19221-s001.pdf]

Supplementary Information

1. Spectral Characterization of 4-(4,5-Diphenyl-1-*p*-tolyl-1*H*-imidazol-2-yl)phenol (8)

Proton Chemical Shift Assignment: The ^{1}H-NMR spectrum (Figure S1) of compound **8** shows singlet at δ 2.26 ppm corresponds to the protons of methyl group at C-13 and the doublet at δ 6.65 ppm corresponds to protons at C-8, 8′ respectively. The multiplet at δ 7.10–7.30 ppm corresponds to the protons at C-11, 11′, 12, 12′, 19, 19′ respectively. A doublet at δ 7.46 ppm corresponds to the protons at C-15, 15′ respectively. The triplets at δ 7.64, δ 7.80 ppm corresponds to the protons at C-17, 21, 16, 16′, 20, 20′ and doublet at δ 7.93 ppm corresponds to the protons at C-7, 7′ respectively and the singlet at δ 9.67 ppm corresponds to the proton (–OH) at C-9.

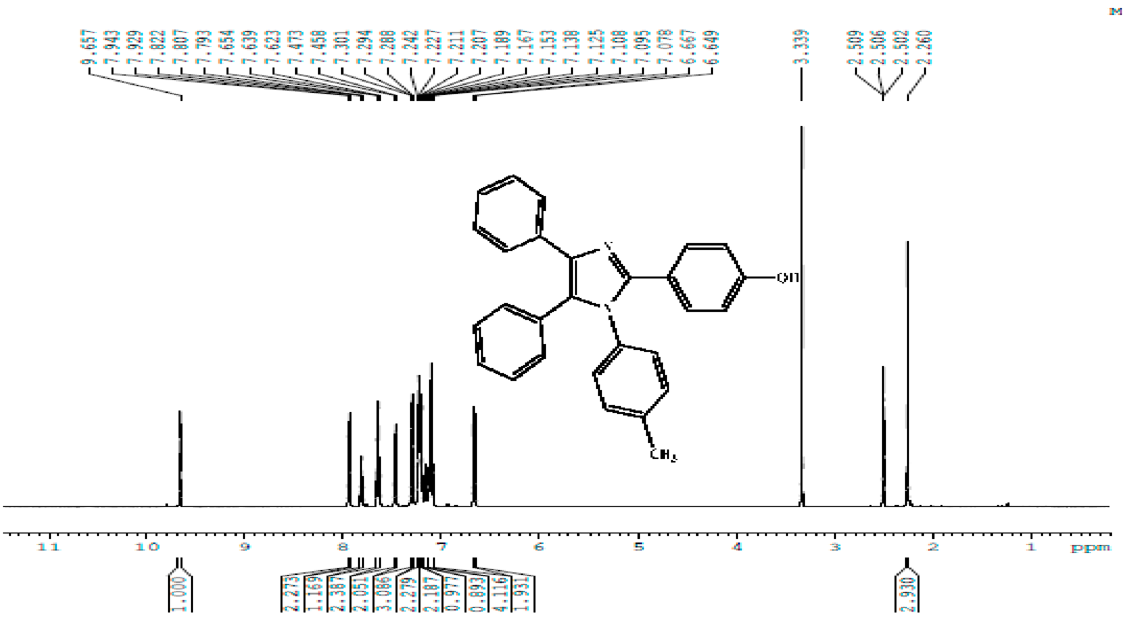

Figure S1. ^{1}H-NMR spectrum of 4-(4,5-diphenyl-1-*p*-tolyl-1*H*-imidazol-2-yl)phenol (**8**).

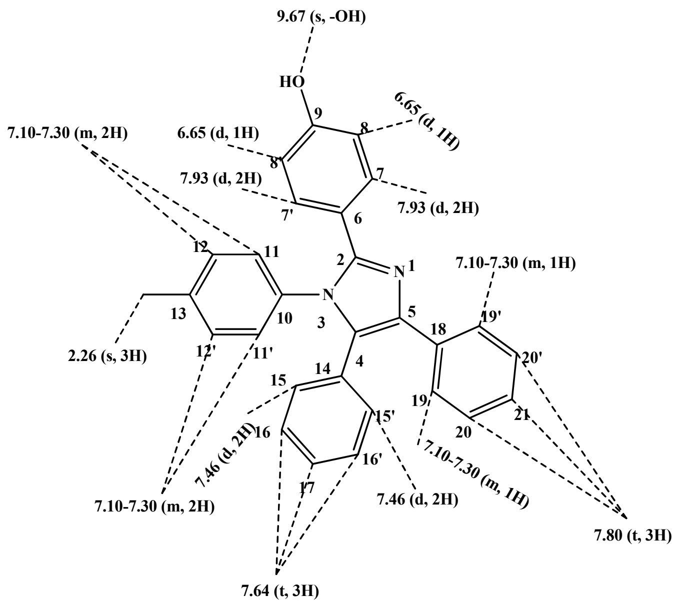

Figure S2. ^{1}H-NMR chemical shift assignment of 4-(4,5-diphenyl-1-*p*-tolyl-1*H*-imidazol-2-yl)phenol (**8**).

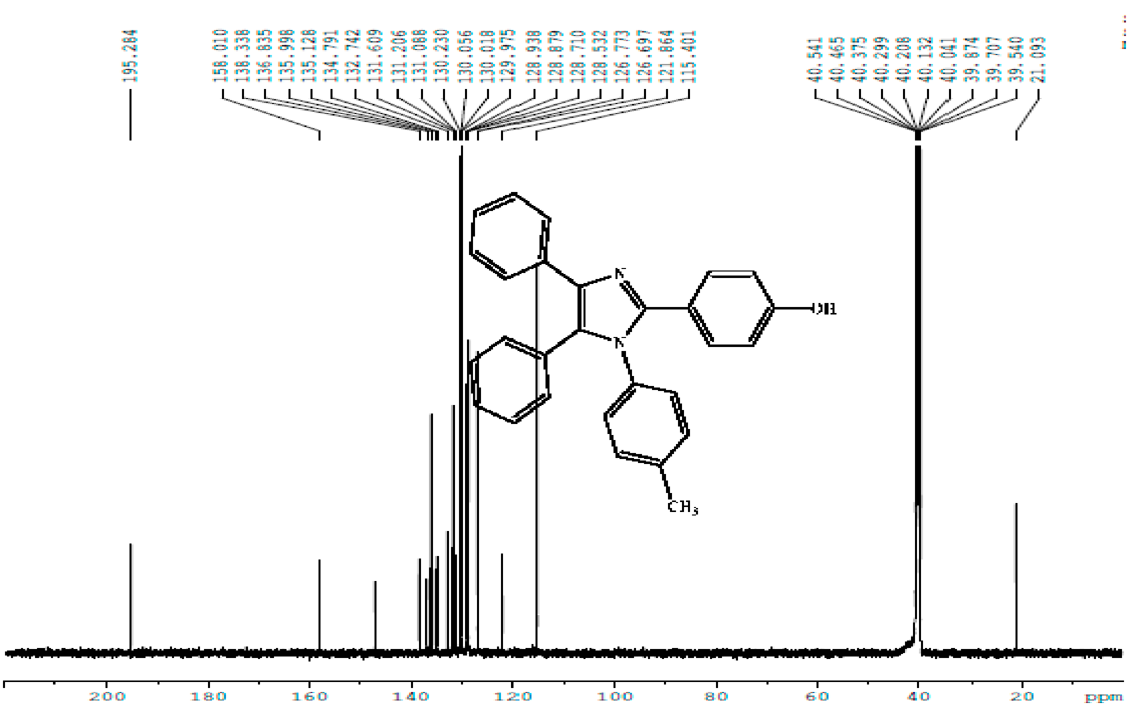

Figure S3. ^{13}C-NMR spectrum of 4-(4,5-diphenyl-1-*p*-tolyl-1*H*-imidazol-2-yl)phenol (**8**).

Carbon Chemical Shift Assignment: The ^{13}C-NMR spectrum (Figure S3) of (**8**). shows chemical shift values at δ 21.09 ppm corresponds to C-13 respectively, and the chemical shift values at δ 115.40, 121.86, 126.69, 126.77, 128.53, 128.71, 128.87, 128.93, 129.97, 130.01, 130.05, 130.23, 131.08, 131.20, 131.60, 132.74, 134.79, 135.12, 135.99, 136.83, 138.33, 158.01 corresponds to the aryl carbons. *m*/*z* value observed at 402.1720 (M^+) peak in HRMS spectra also confirms the formation of target molecule.

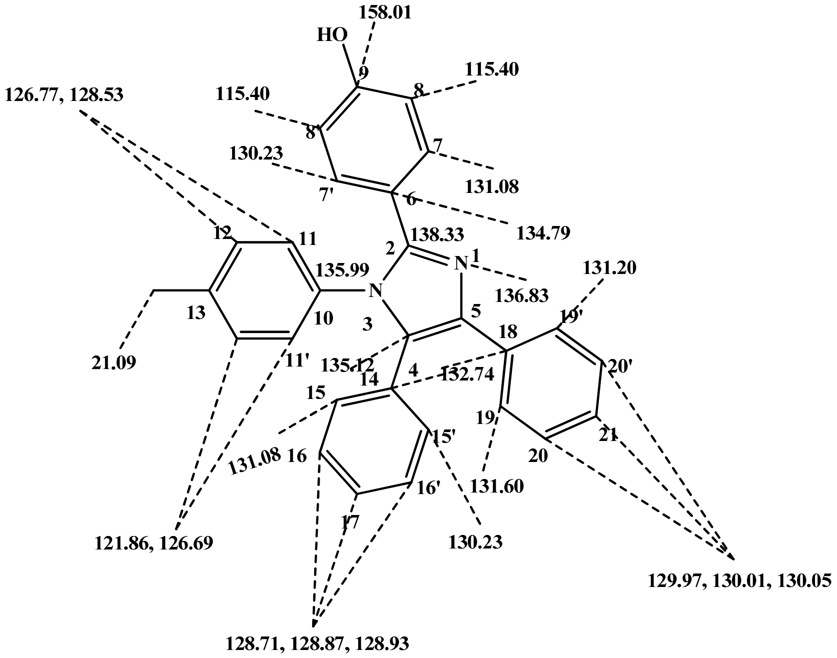

Figure S4. ^{13}C-NMR chemical shift assignment of 4-(4,5-diphenyl-1-*p*-tolyl-1*H*-imidazol-2-yl)phenol (**8**).

2. Spectral Evidences

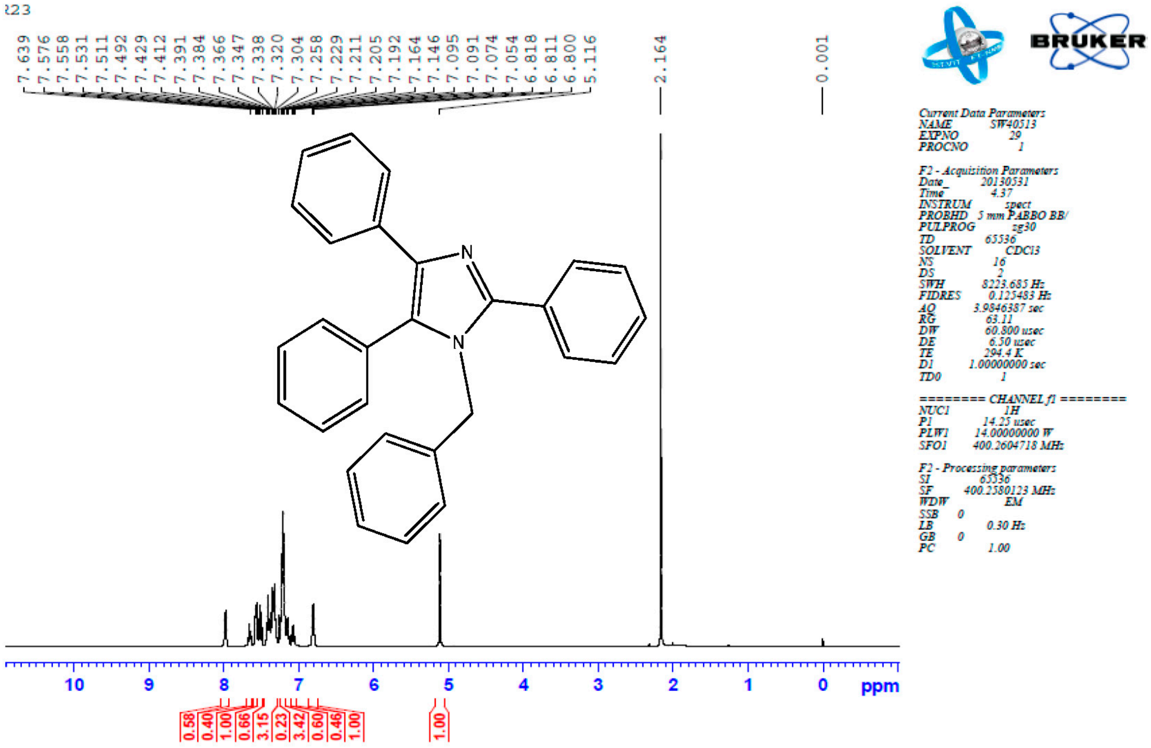

Figure S5. ^{1}H-NMR spectrum of 1-benzyl-2,4,5-triphenyl-1*H*-imidazole (**1**).

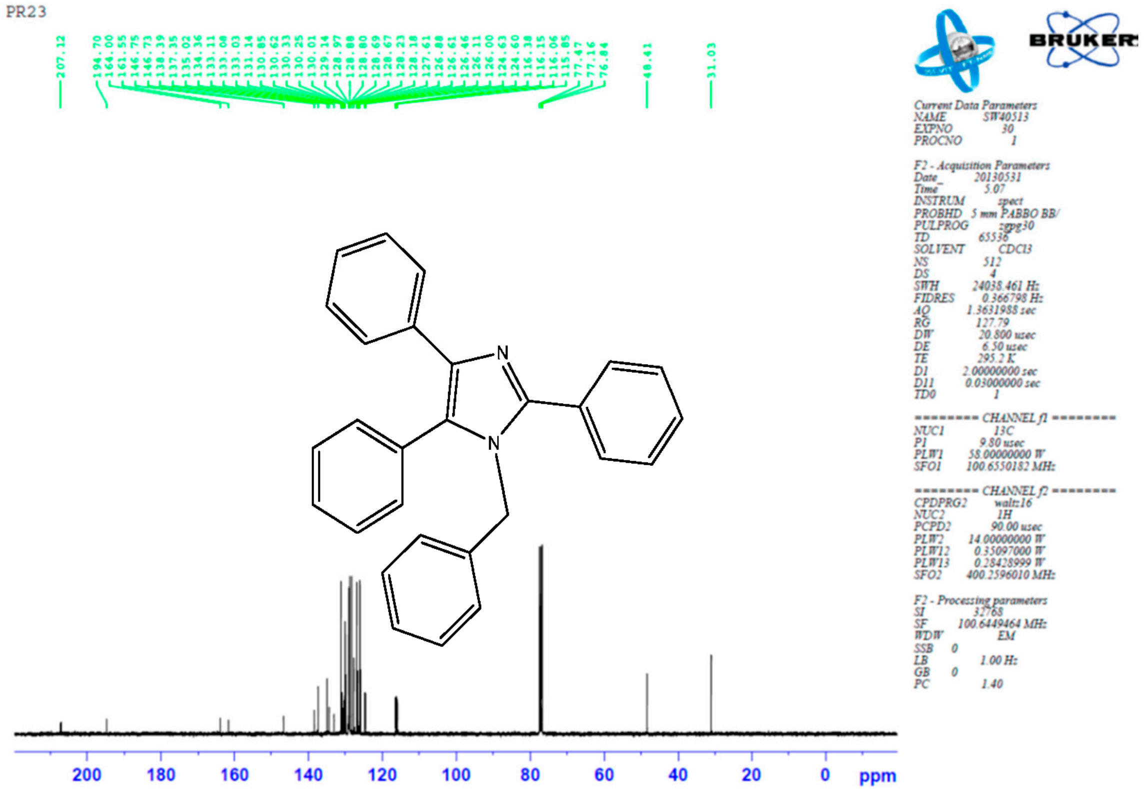

Figure S6. ^{13}C-NMR spectrum of 1-benzyl-2,4,5-triphenyl-1*H*-imidazole (**1**).

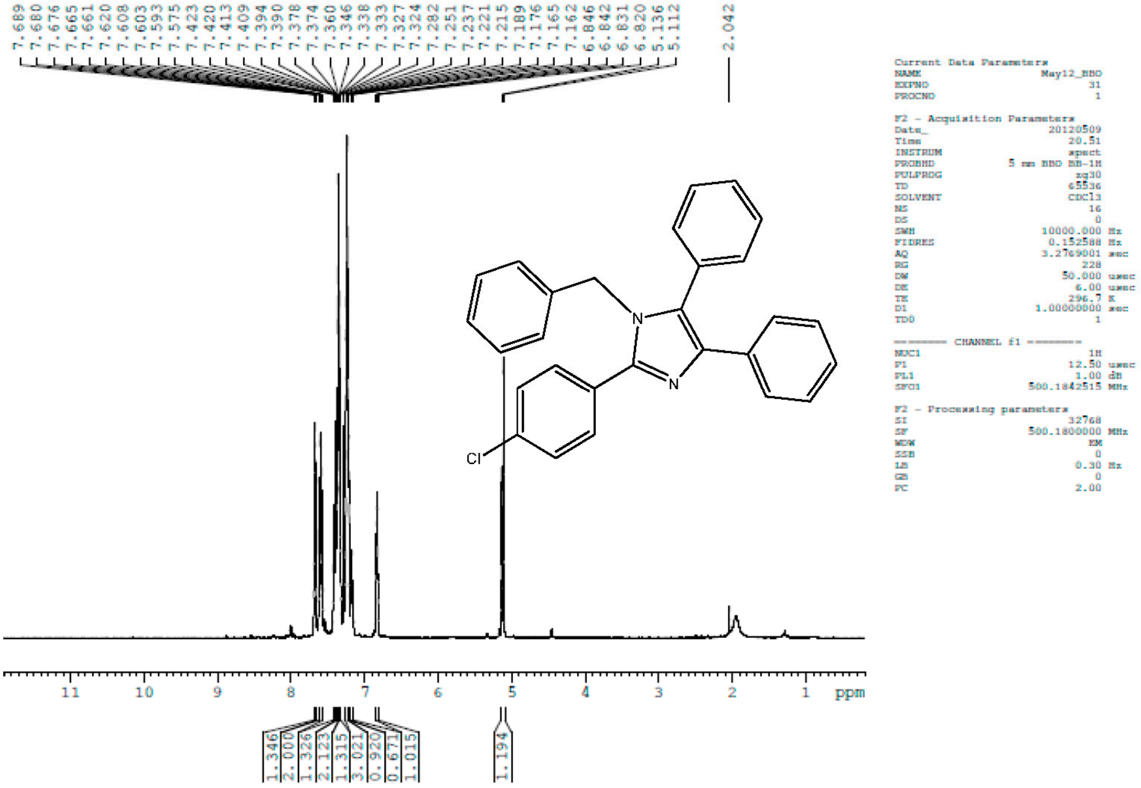

Figure S7. ^{1}H-NMR spectrum of 1-benzyl-2-(4-chlorophenyl)-4,5-diphenyl-1*H*-imidazole (**2**).

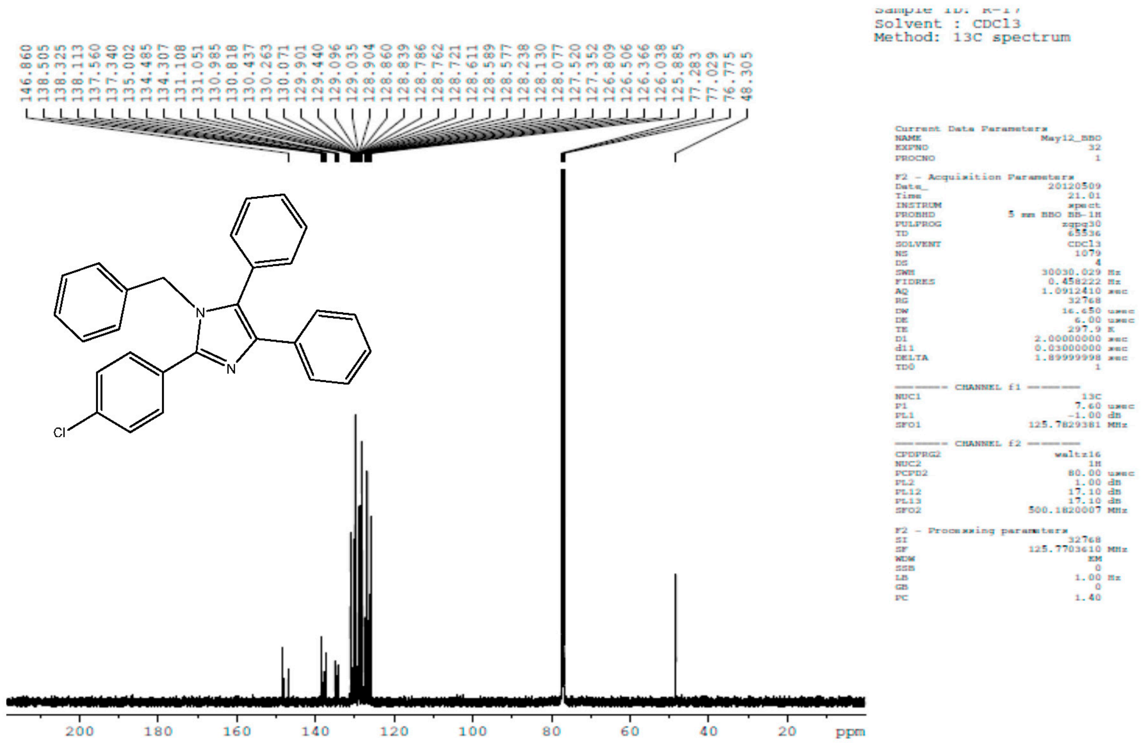

Figure S8. ^{13}C-NMR spectrum of 1-benzyl-2-(4-chlorophenyl)-4,5-diphenyl-1*H*-imidazole (**2**).

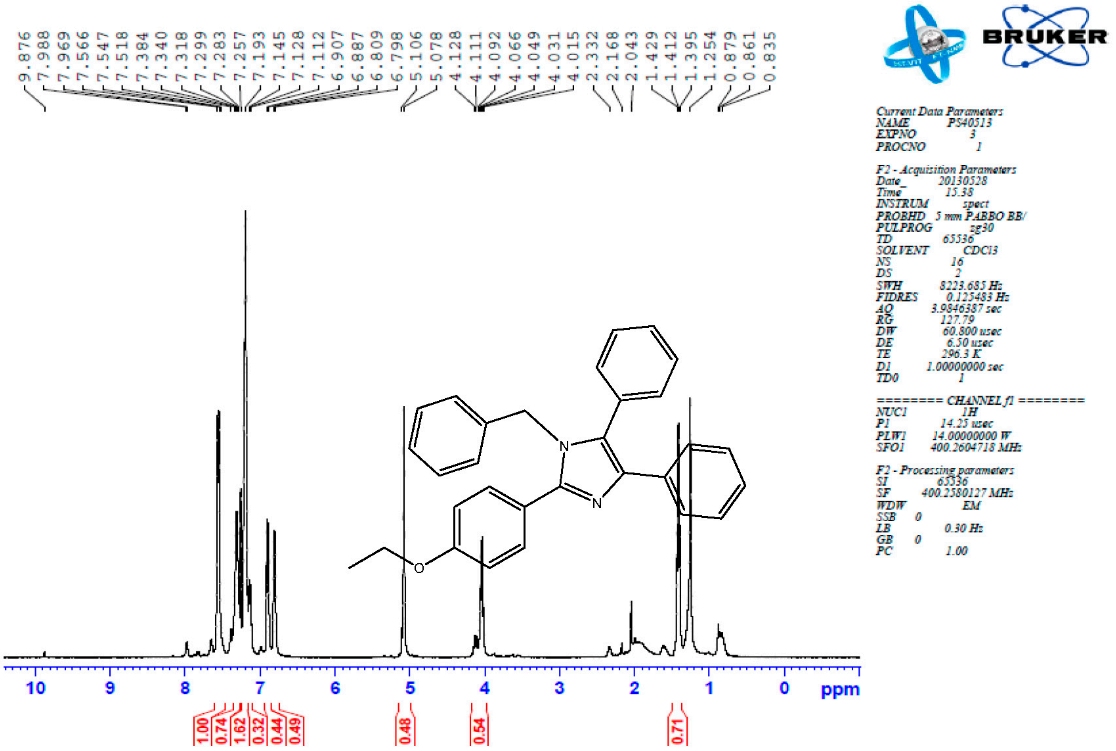

Figure S9. ^{1}H-NMR spectrum of 1-benzyl-2-(4-ethoxyphenyl)-4,5-diphenyl-1*H*-imidazole (**3**).

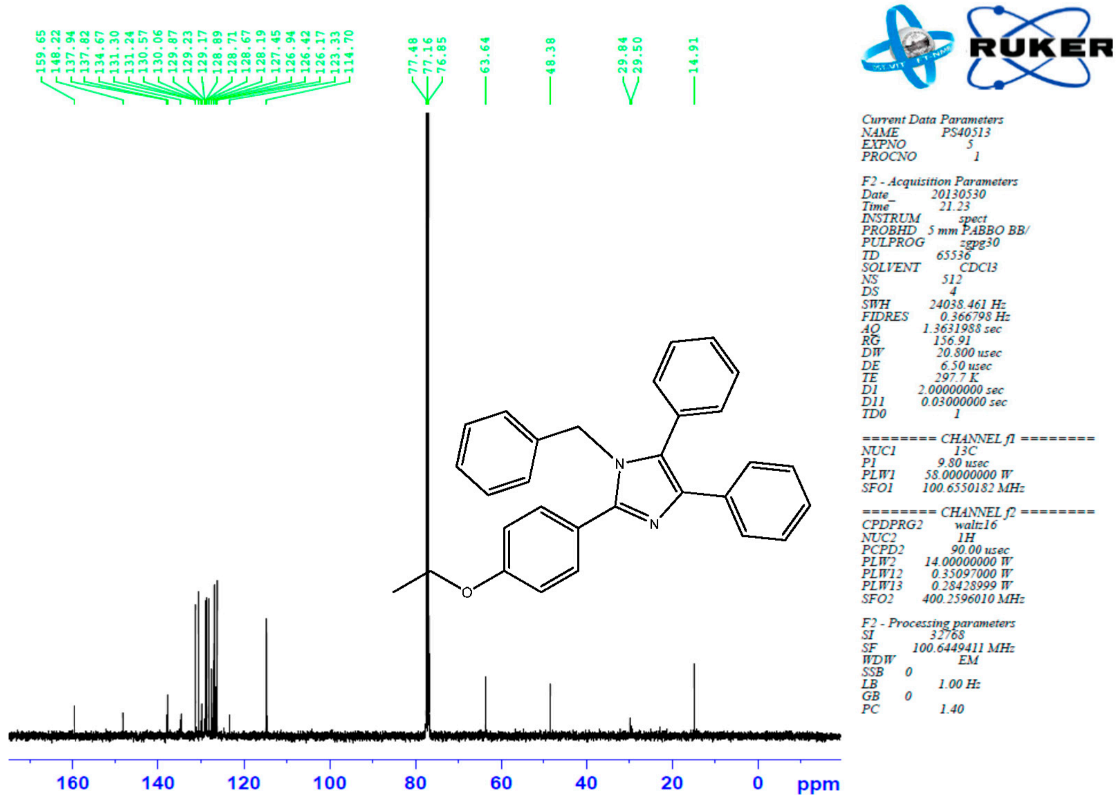

Figure S10. ^{1}H-NMR spectrum of 1-benzyl-2-(4-ethoxyphenyl)-4,5-diphenyl-1*H*-imidazole (**3**).

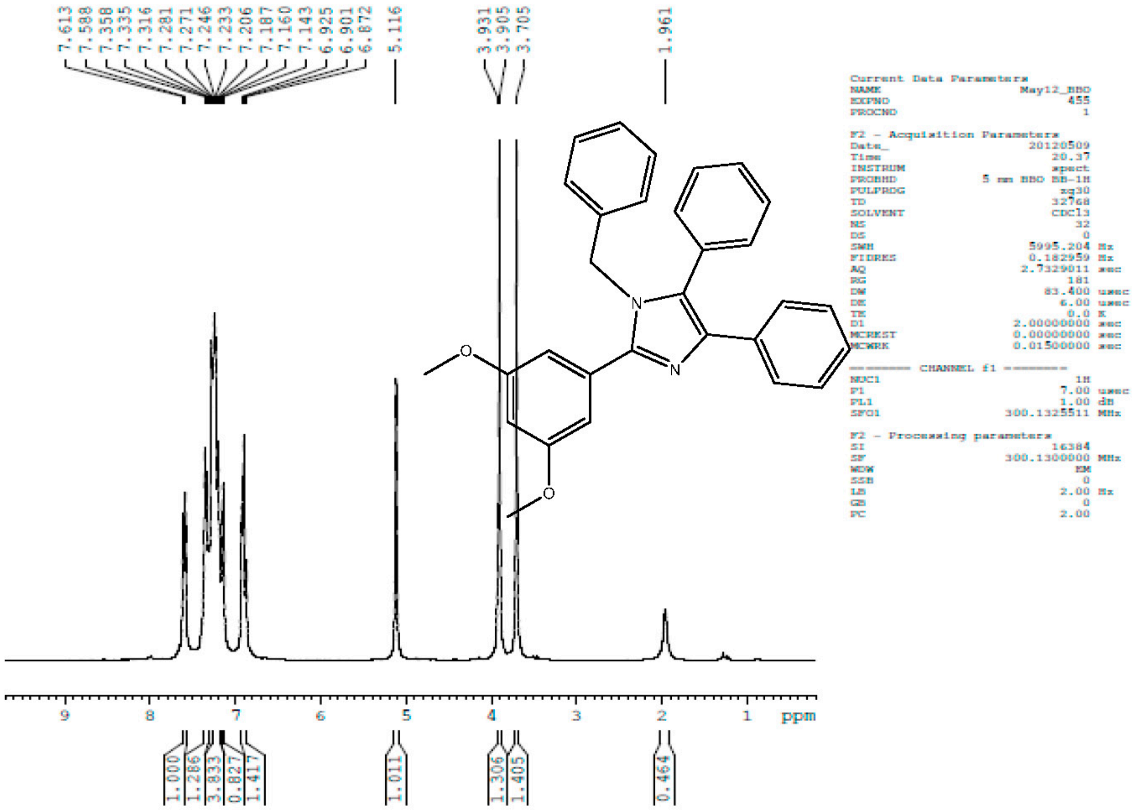

Figure S11. ^{1}H-NMR spectrum of 1-benzyl-2-(3,5-dimethoxyphenyl)-4,5-diphenyl-1*H*-imidazole (**4**).

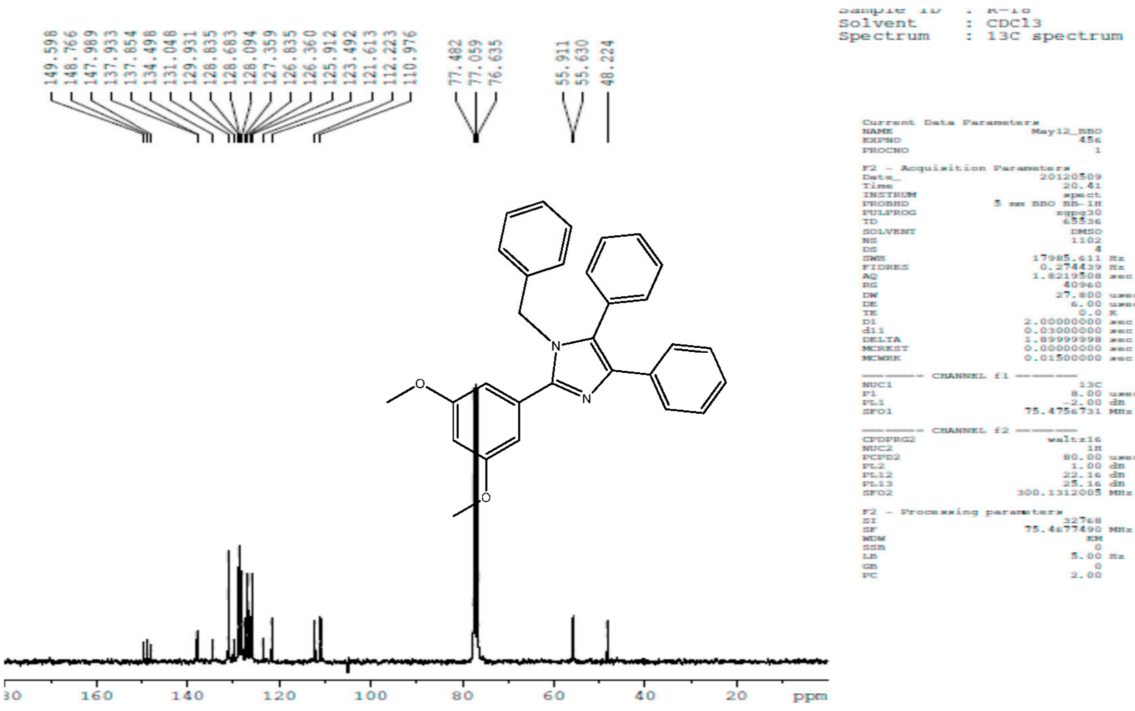

Figure S12. ^{1}H-NMR spectrum of 1-benzyl-2-(3,5-dimethoxyphenyl)-4,5-diphenyl-1*H*-imidazole (**4**).

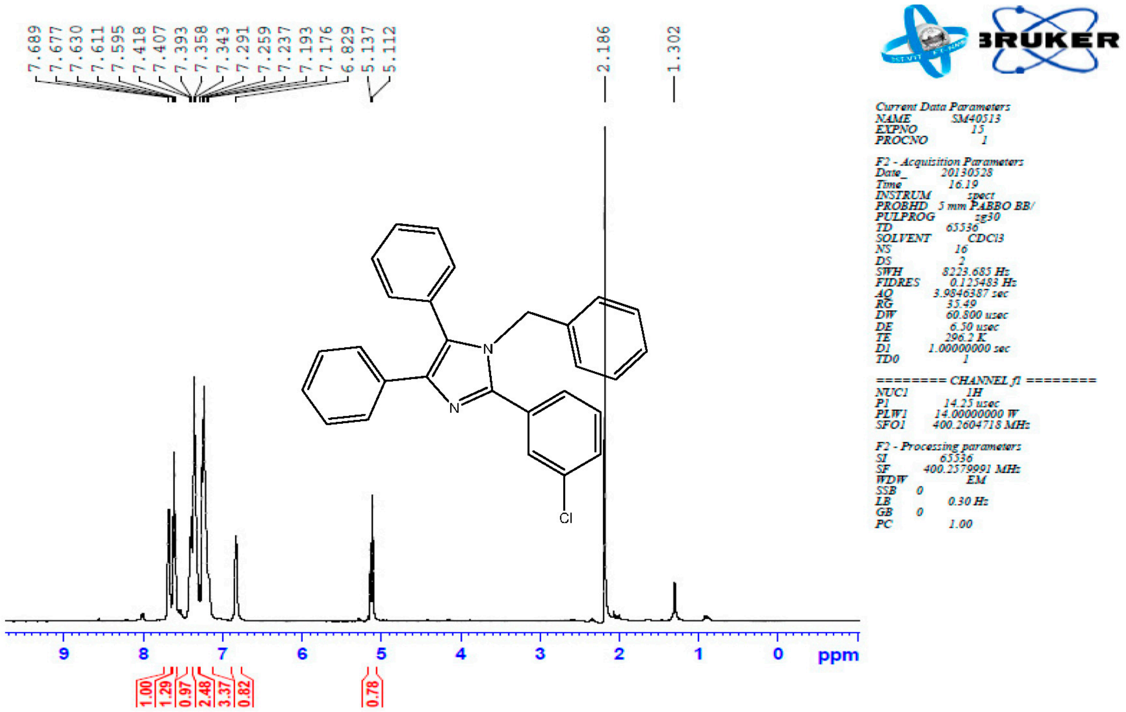

Figure S13. ¹H-NMR spectrum of 1-benzyl-2-(3-chlorophenyl)-4,5-diphenyl-1*H*-imidazole (**5**).

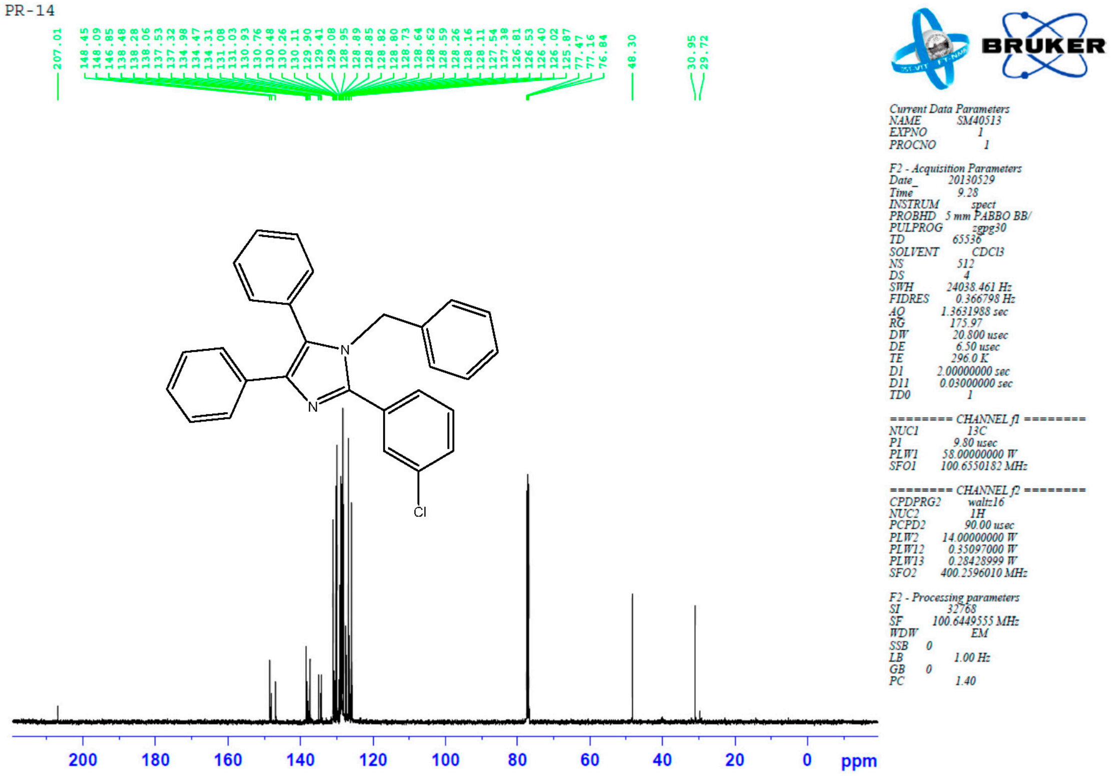

Figure S14. ¹³C-NMR spectrum of 1-benzyl-2-(3-chlorophenyl)-4,5-diphenyl-1*H*-imidazole (**5**).

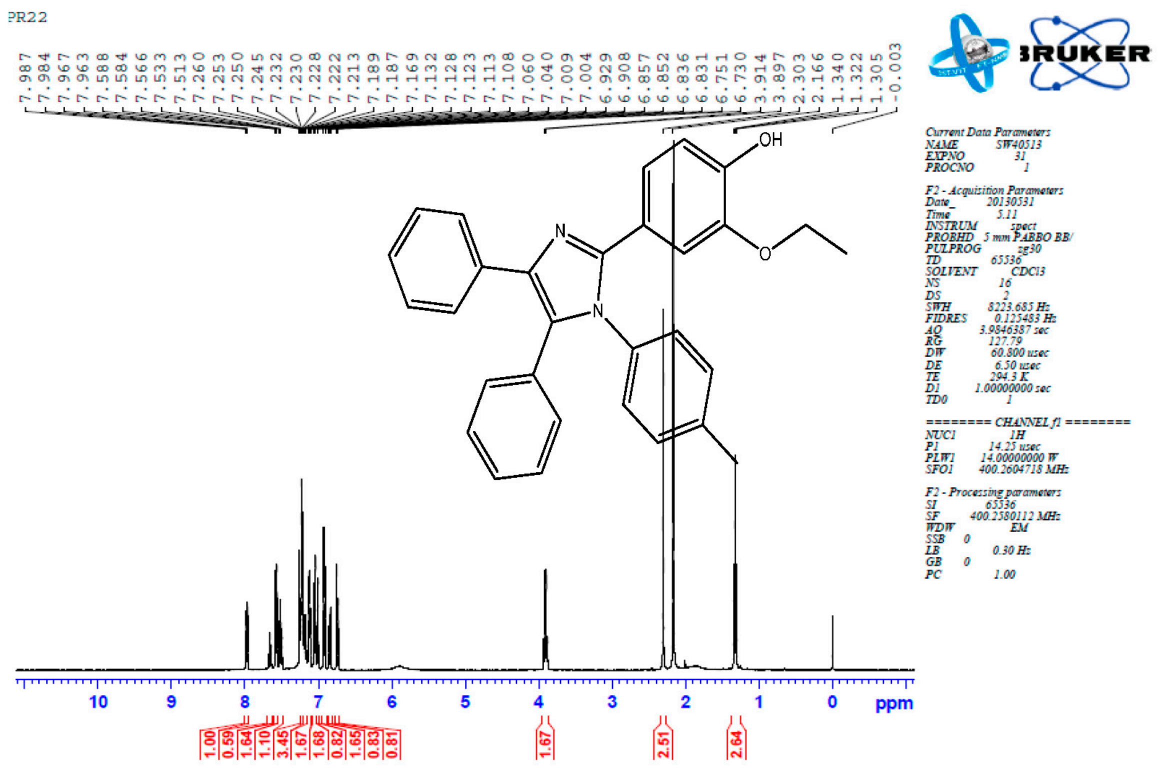

Figure S15. ^{1}H-NMR spectrum of 4-(4,5-diphenyl-1-*p*-tolyl-1*H*-imidazol-2-yl)-2-ethoxyphenol (**6**).

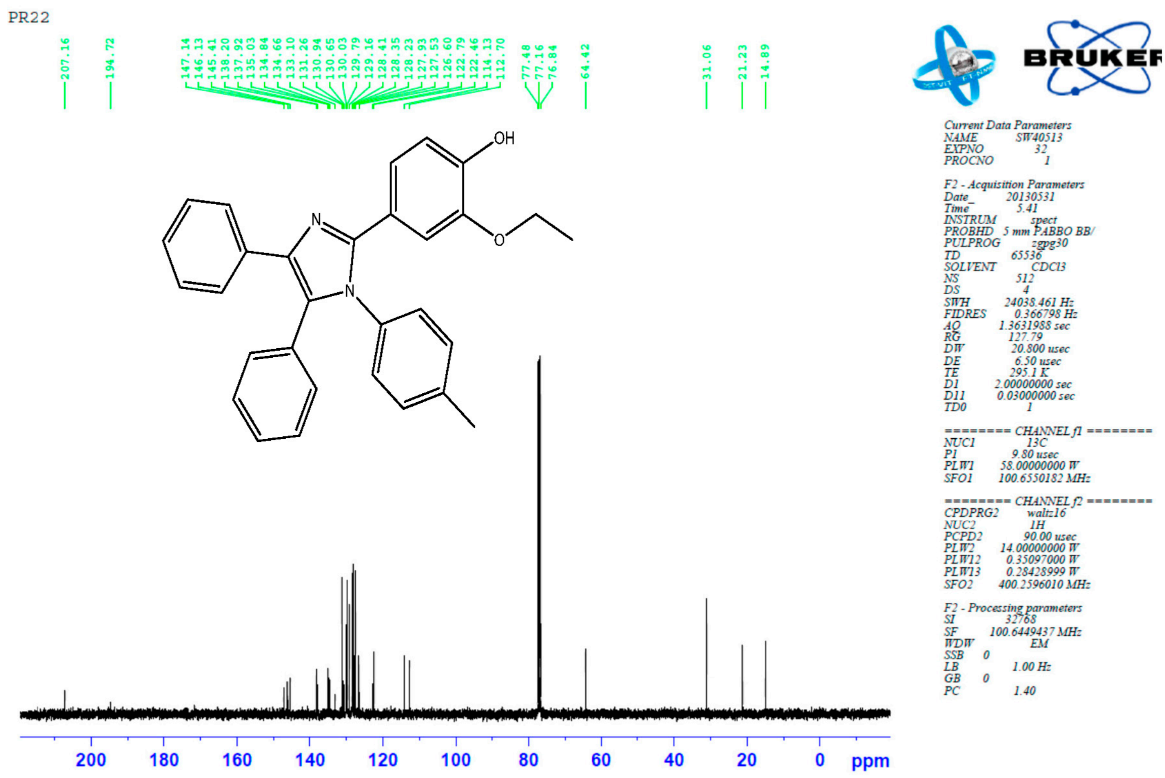

Figure S16. ^{13}C-NMR spectrum of 4-(4,5-diphenyl-1-*p*-tolyl-1*H*-imidazol-2-yl)-2-ethoxyphenol (**6**).

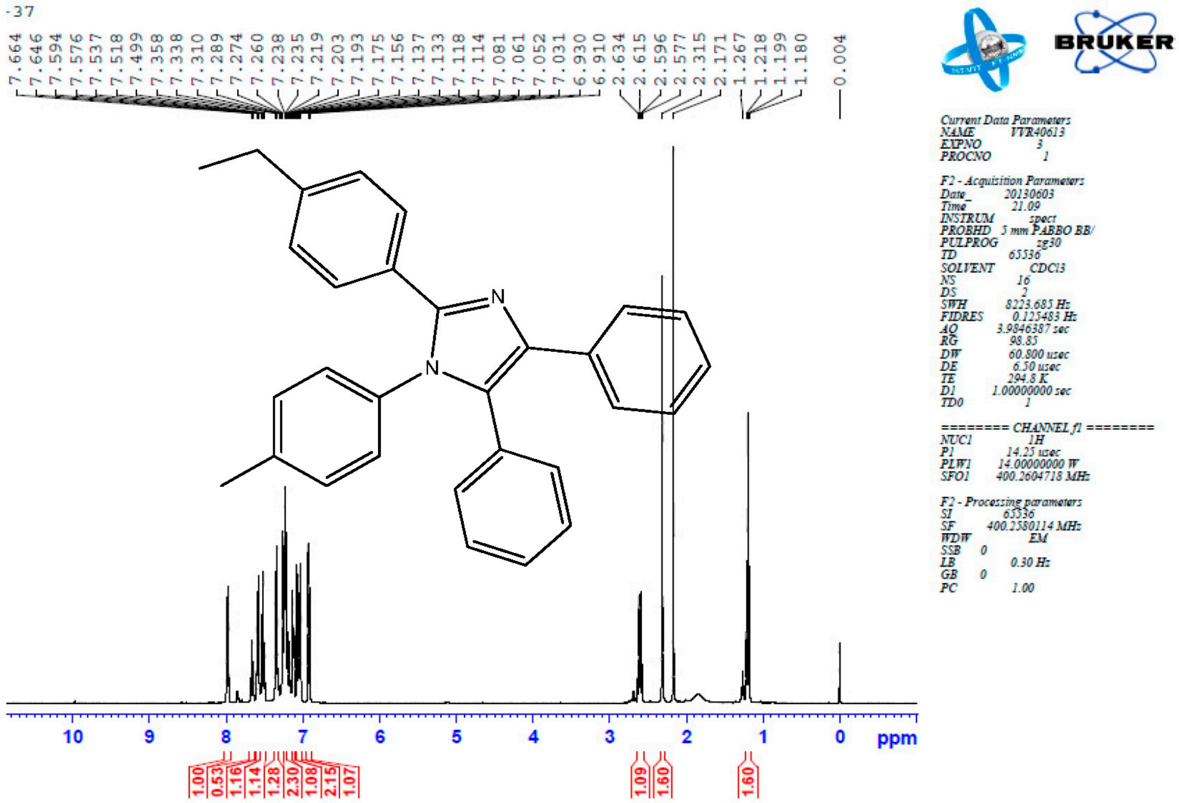

Figure S17. ^{1}H-NMR spectrum of 2-(4-ethylphenyl)-4,5-diphenyl-1-(*p*-tolyl)-1*H*-imidazole (**7**).

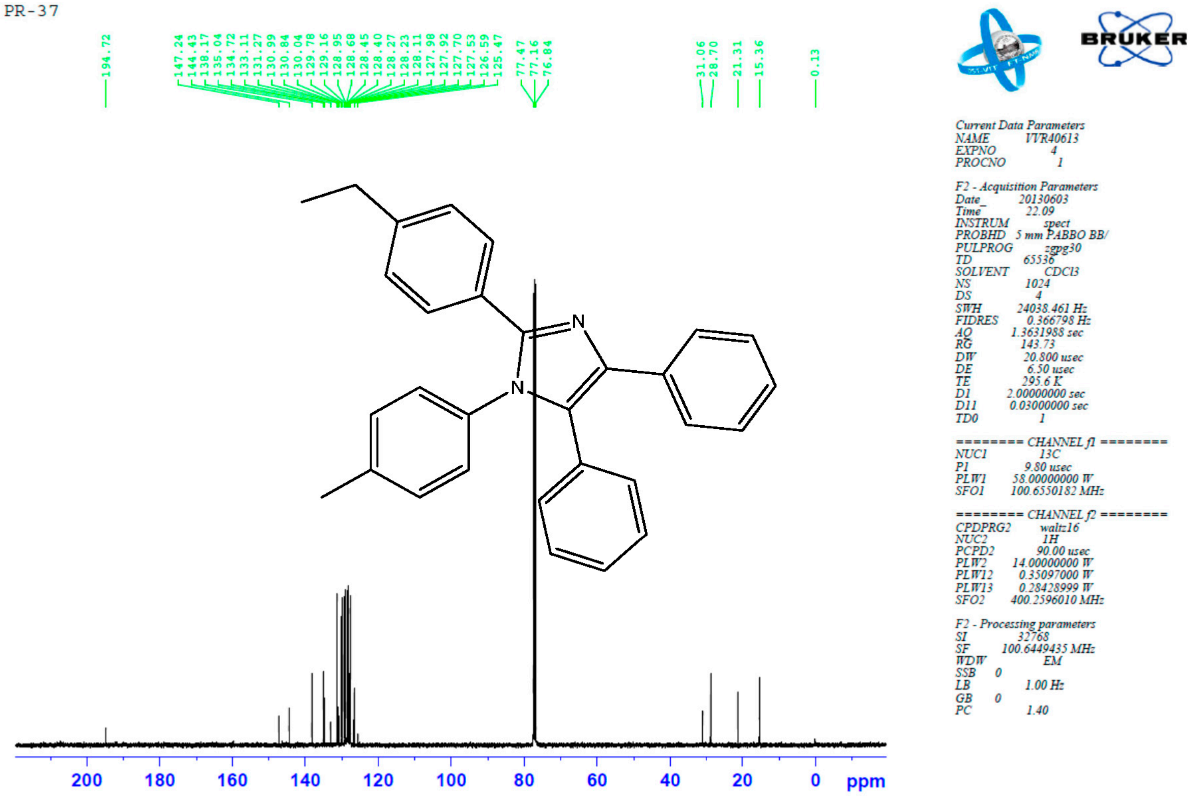

Figure S18. ^{13}C-NMR spectrum of 2-(4-ethylphenyl)-4,5-diphenyl-1-(*p*-tolyl)-1*H*-imidazole (**7**).

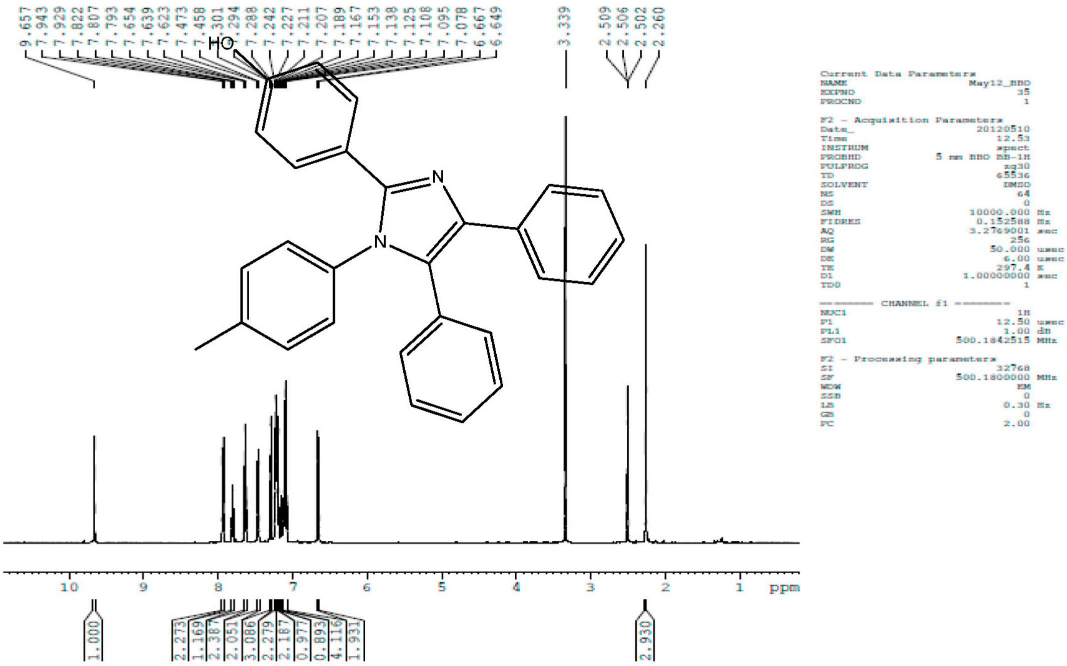

Figure S19. ^{1}H-NMR spectrum of 4-(4,5-diphenyl-1-(*p*-tolyl)-1*H*-imidazol-2-yl)phenol (**8**).

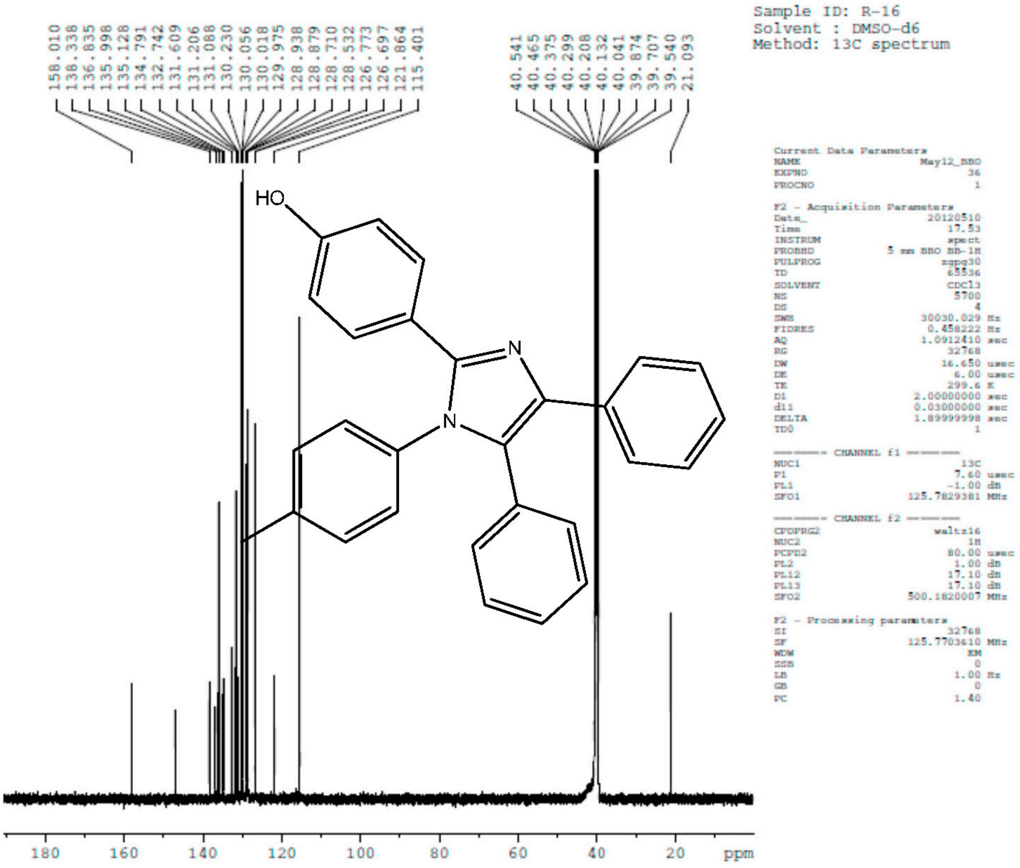

Figure S20. ^{13}C-NMR spectrum of 4-(4,5-diphenyl-1-(*p*-tolyl)-1*H*-imidazol-2-yl)phenol (**8**).

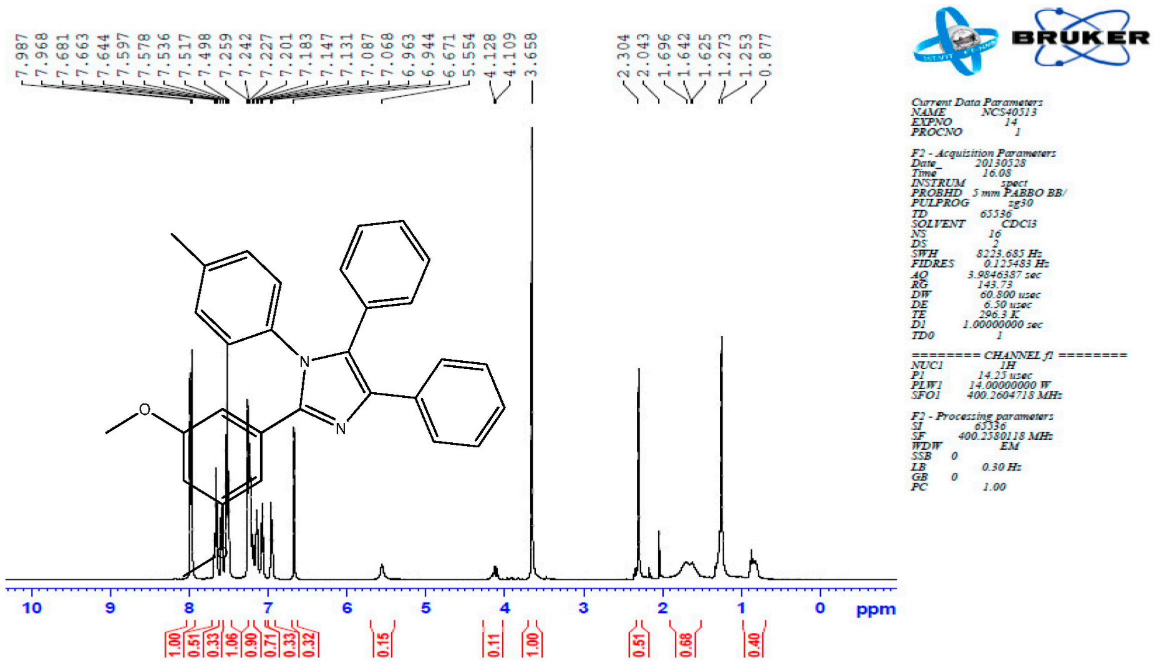

Figure S21. ^{1}H-NMR spectrum of 2-(3,5-dimethoxyphenyl)-4,5-diphenyl-1-(*p*-tolyl)-1*H*-imidazole (**9**).

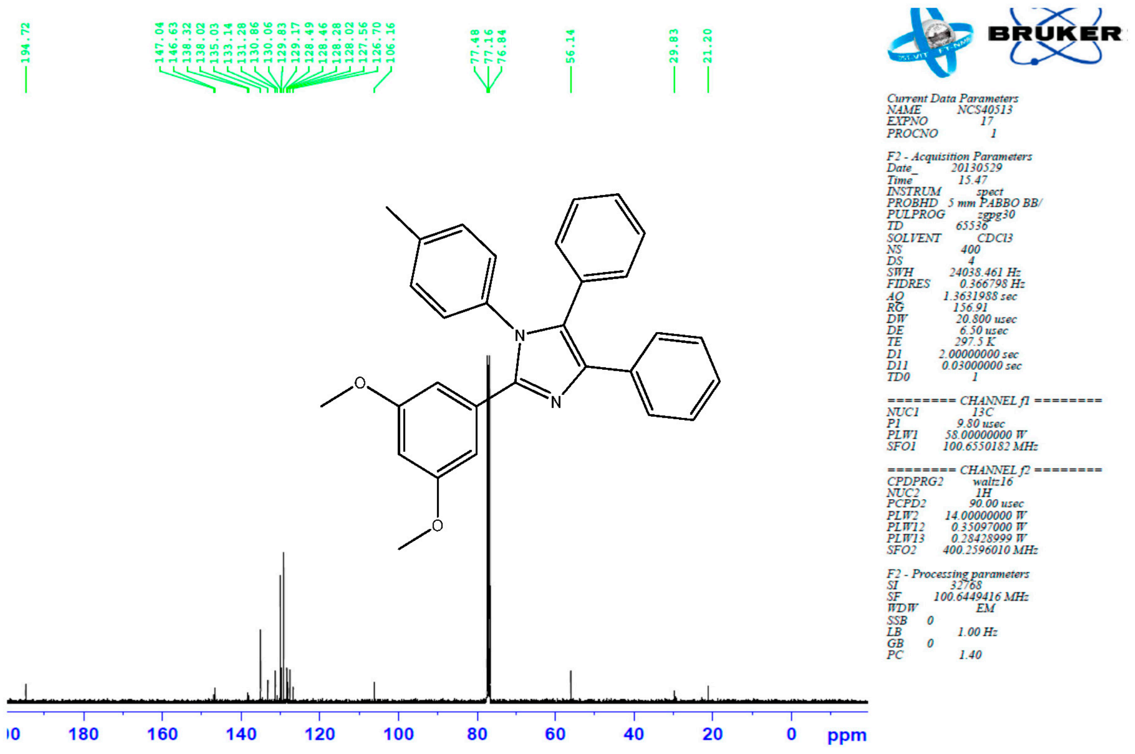

Figure S22. ^{13}C-NMR spectrum of 2-(3,5-dimethoxyphenyl)-4,5-diphenyl-1-(*p*-tolyl)-1*H*-imidazole (**9**).

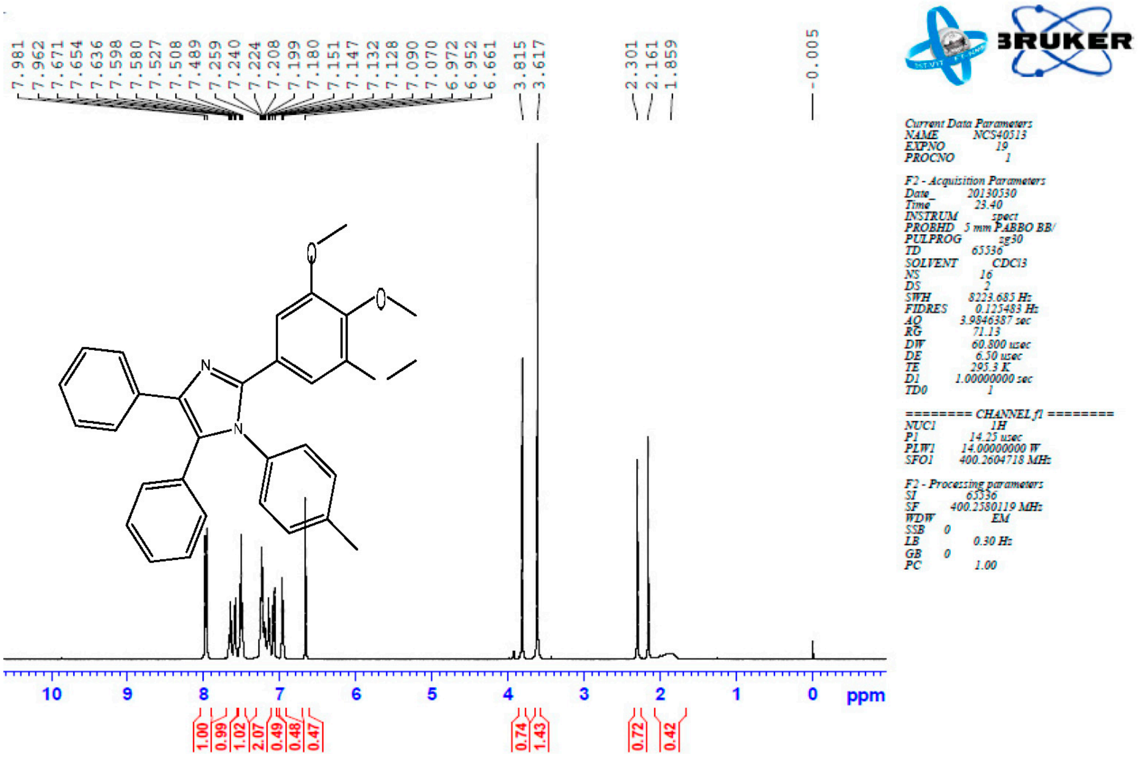

Figure S23. ^{1}H-NMR spectrum of 4,5-diphenyl-1-(*p*-tolyl)-2-(3,4,5-trimethoxyphenyl)-1*H*-imidazole (**10**).

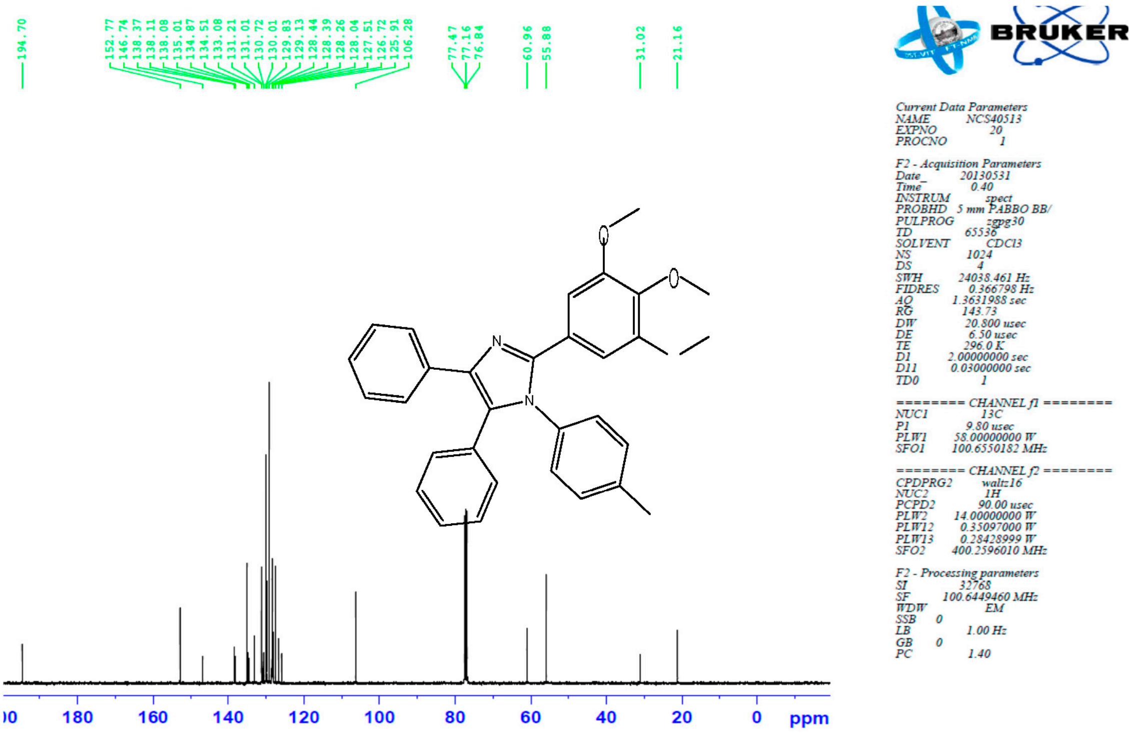

Figure S24. ^{13}C-NMR spectrum of 4,5-diphenyl-1-(*p*-tolyl)-2-(3,4,5-trimethoxyphenyl)-1*H*-imidazole (**10**).

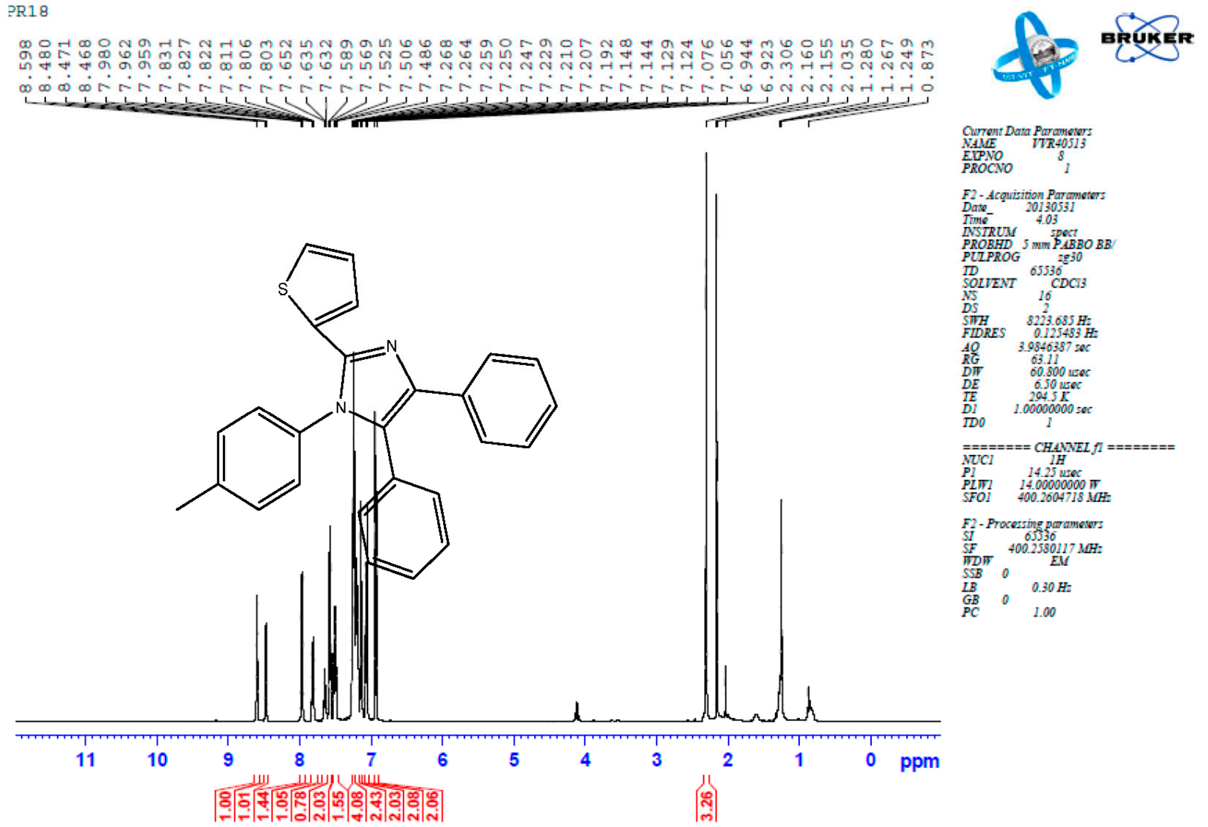

Figure S25. ^{1}H-NMR spectrum of 4,5-diphenyl-2-(thiophen-2-yl)-1-(*p*-tolyl)-1*H*-imidazole (**11**).

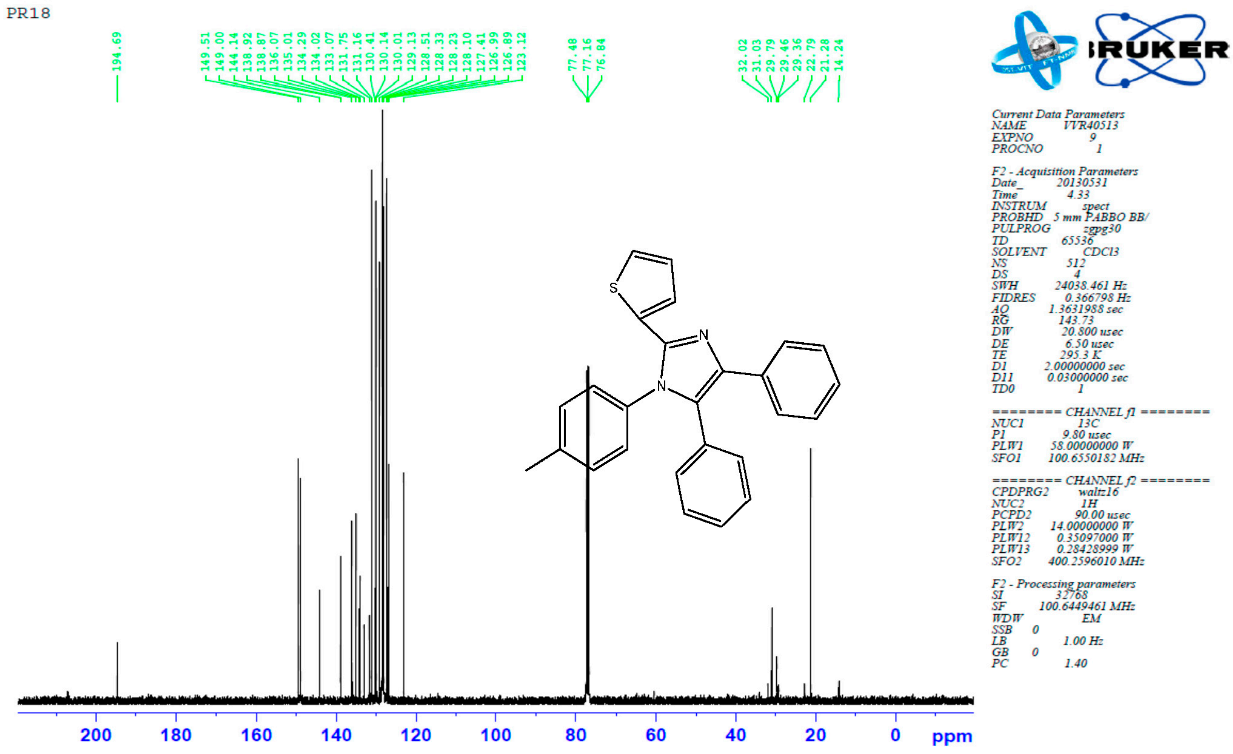

Figure S26. ^{13}C-NMR spectrum of 4,5-diphenyl-2-(thiophen-2-yl)-1-(*p*-tolyl)-1*H*-imidazole (**11**).

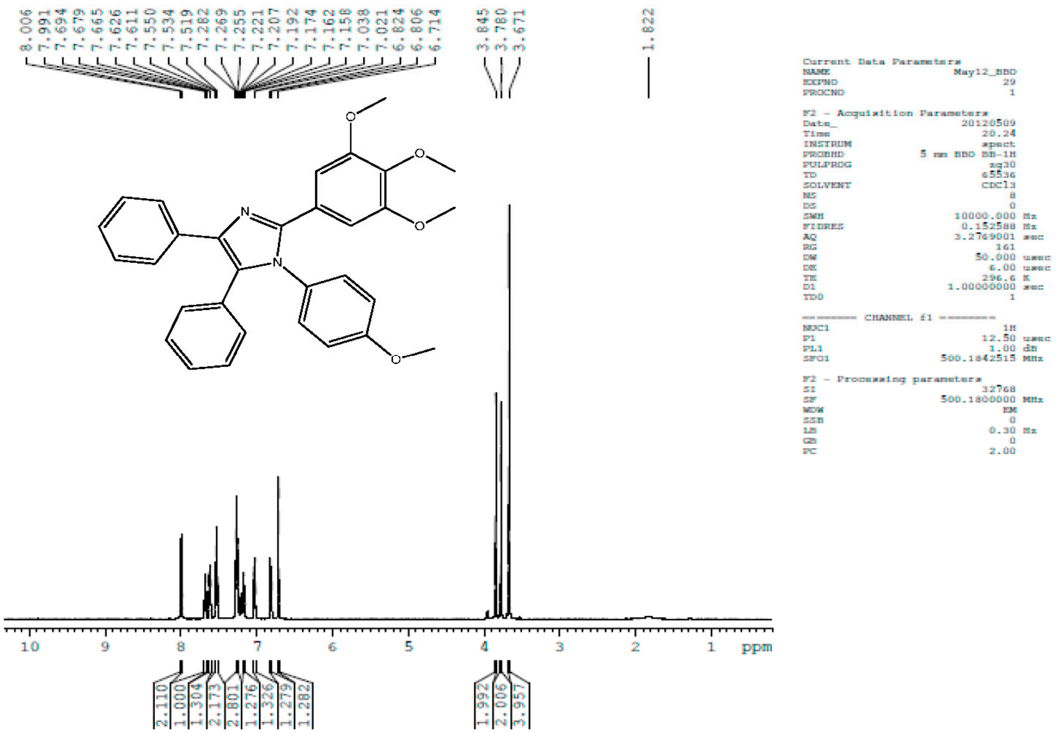

Figure S27. ^{1}H-NMR spectrum of 1-(4-methoxyphenyl)-4,5-diphenyl-2-(3,4,5-trimethoxyphenyl)-1*H*-imidazole (**12**).

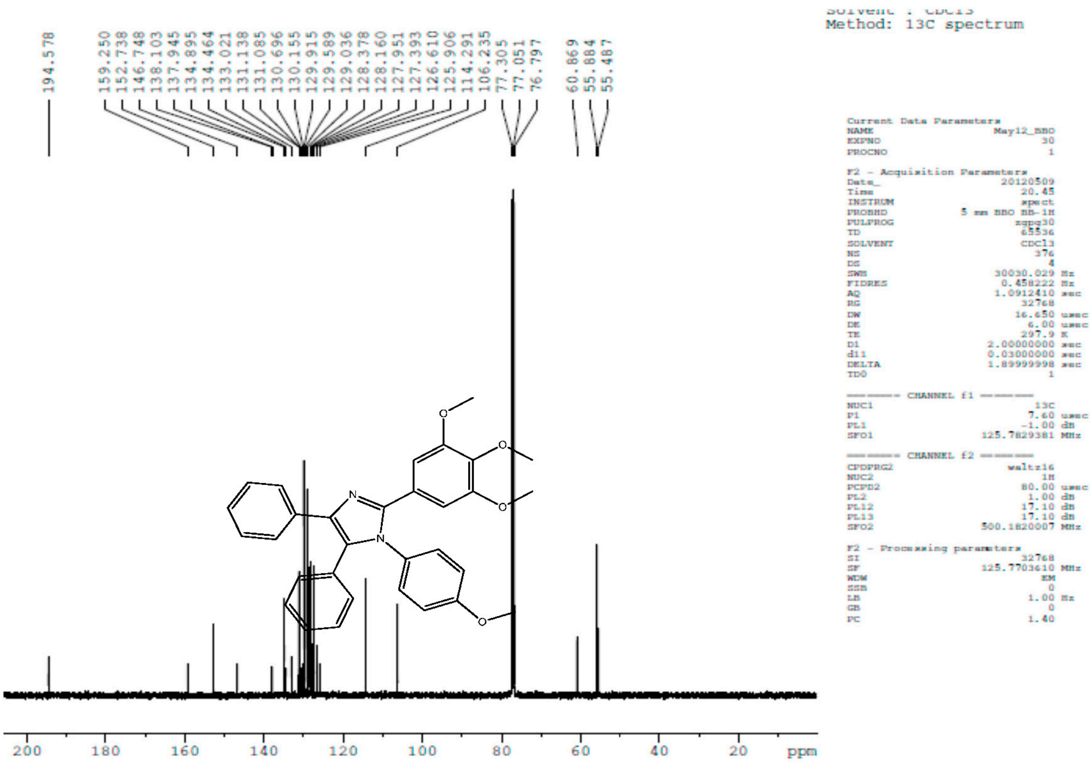

Figure S28. ^{13}C-NMR spectrum of 1-(4-methoxyphenyl)-4,5-diphenyl-2-(3,4,5-trimethoxyphenyl)-1*H*-imidazole (**12**).

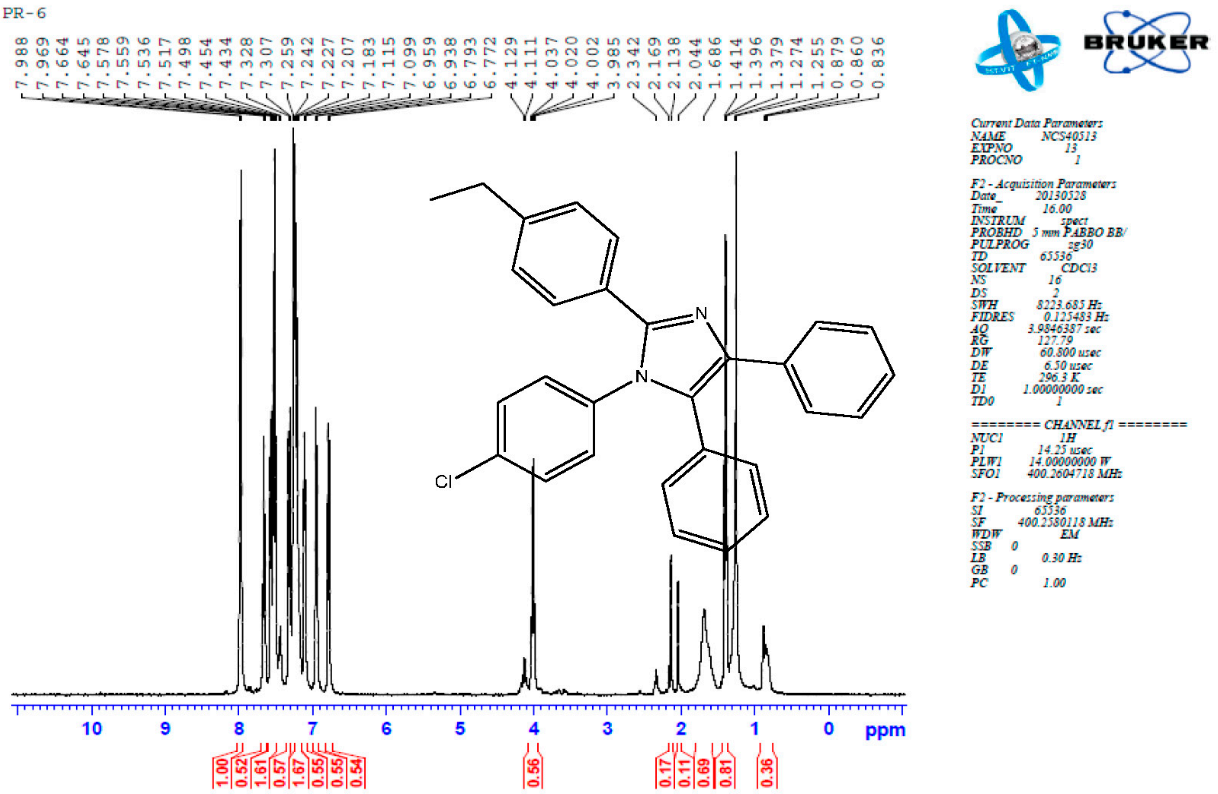

Figure S29. ^{1}H-NMR spectrum of 1-(4-chlorophenyl)-2-(4-ethylphenyl)-4,5-diphenyl-1*H*-imidazole (**13**).

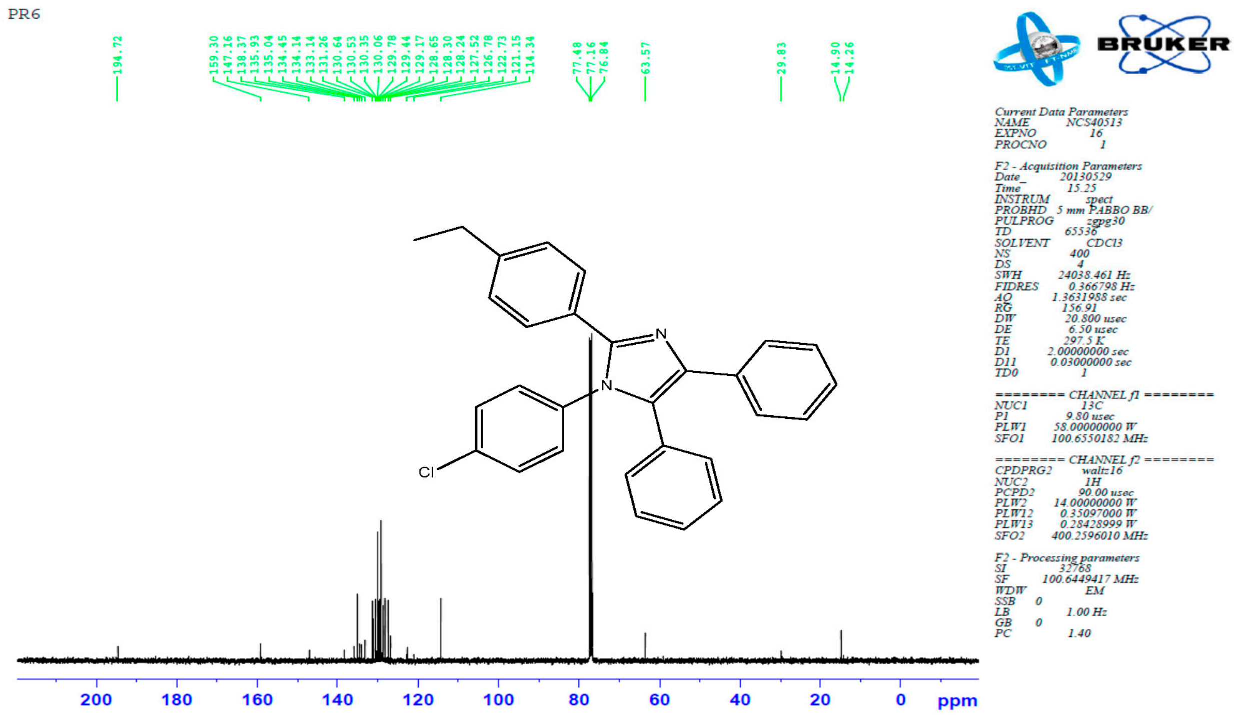

Figure S30. ^{13}C-NMR spectrum of 1-(4-chlorophenyl)-2-(4-ethylphenyl)-4,5-diphenyl-1*H*-imidazole (**13**).

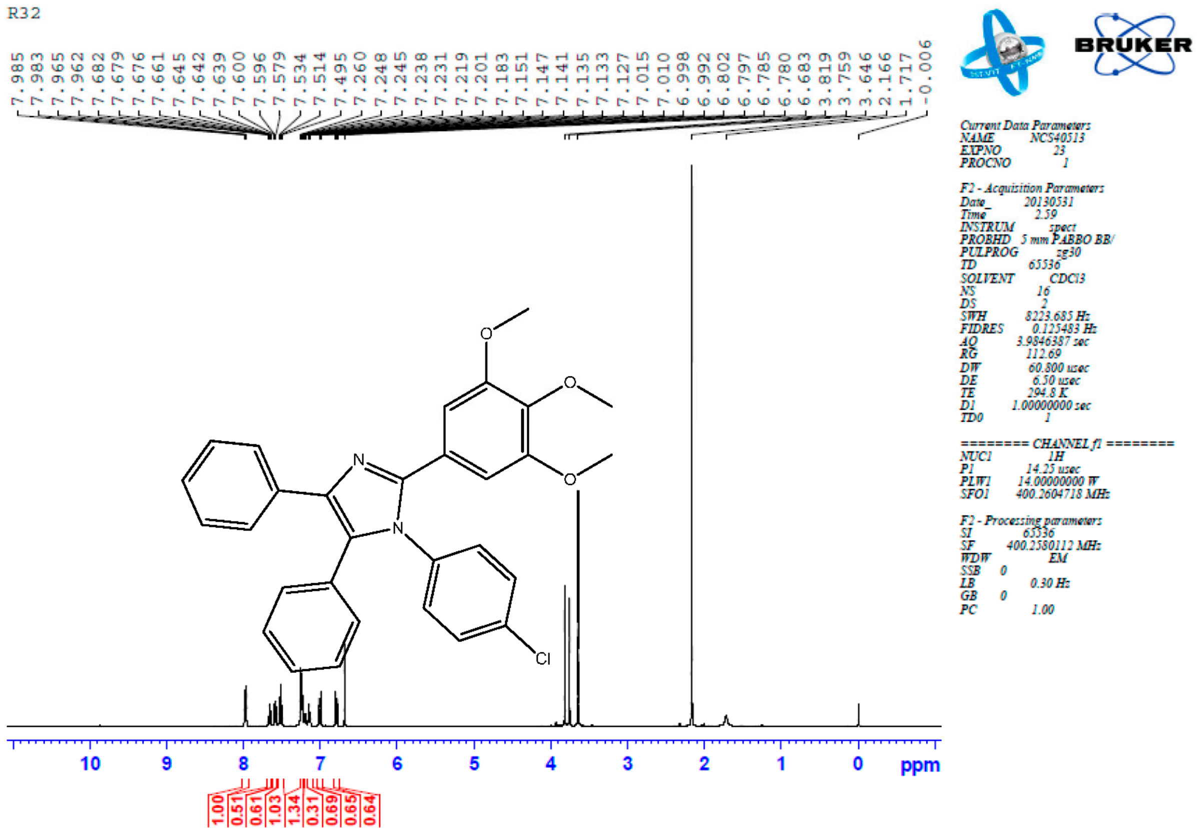

Figure S31. ^{1}H-NMR spectrum of 1-(4-chlorophenyl)-4,5-diphenyl-2-(3,4,5-trimethoxyphenyl)-1*H*-imidazole (**14**).

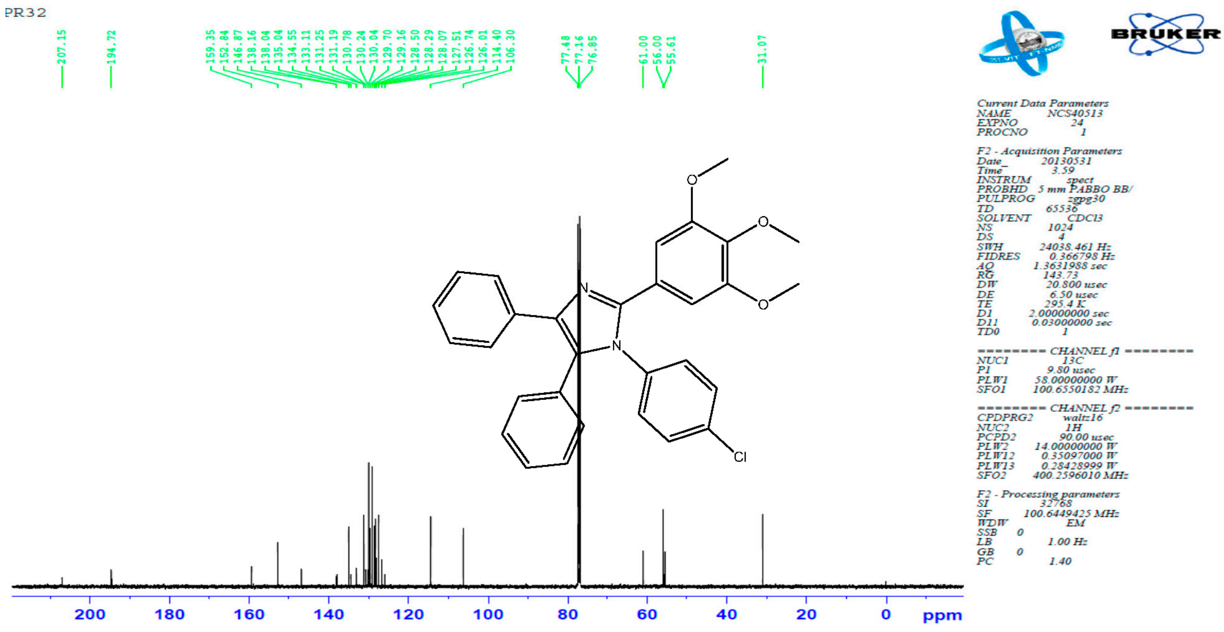

Figure S32. ^{13}C-NMR spectrum of 1-(4-chlorophenyl)-4,5-diphenyl-2-(3,4,5-trimethoxyphenyl)-1*H*-imidazole (**14**).

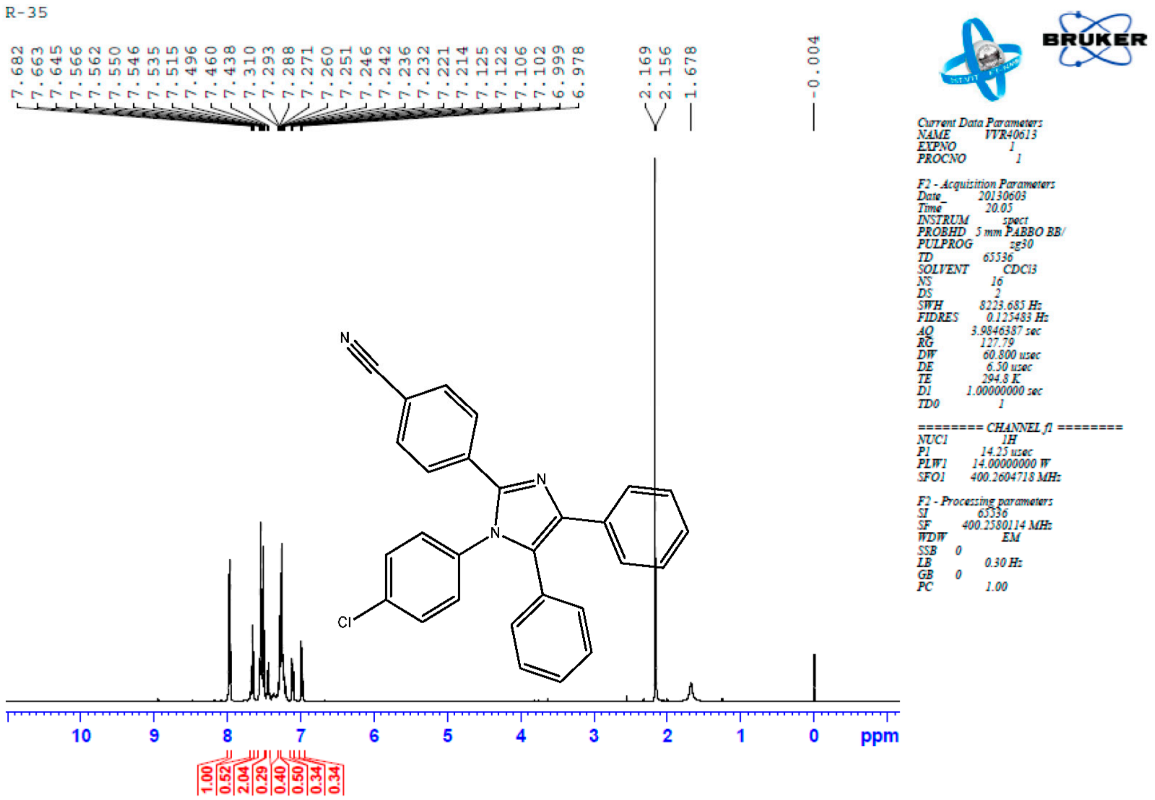

Figure S33. ^{1}H-NMR spectrum of 4-(1-(4-chlorophenyl)-4,5-diphenyl-1*H*-imidazol-2-yl) benzonitrile (**15**).

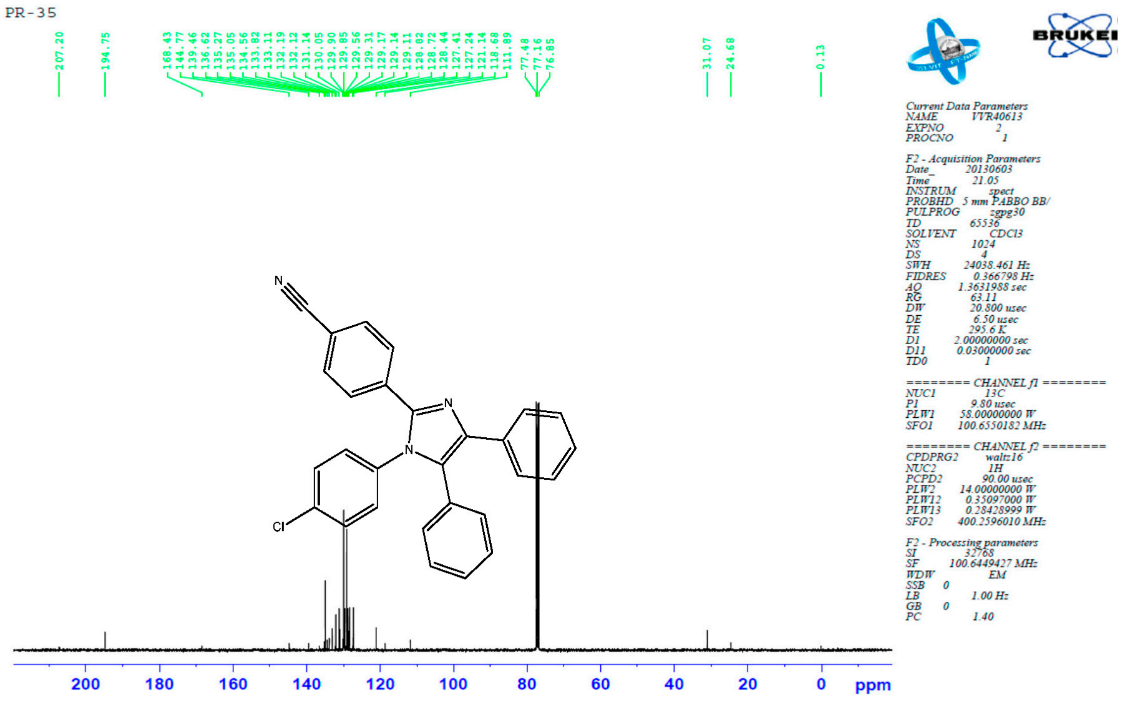

Figure S34. ^{13}C-NMR spectrum of 4-(1-(4-chlorophenyl)-4,5-diphenyl-1*H*-imidazol-2-yl) benzonitrile (**15**).

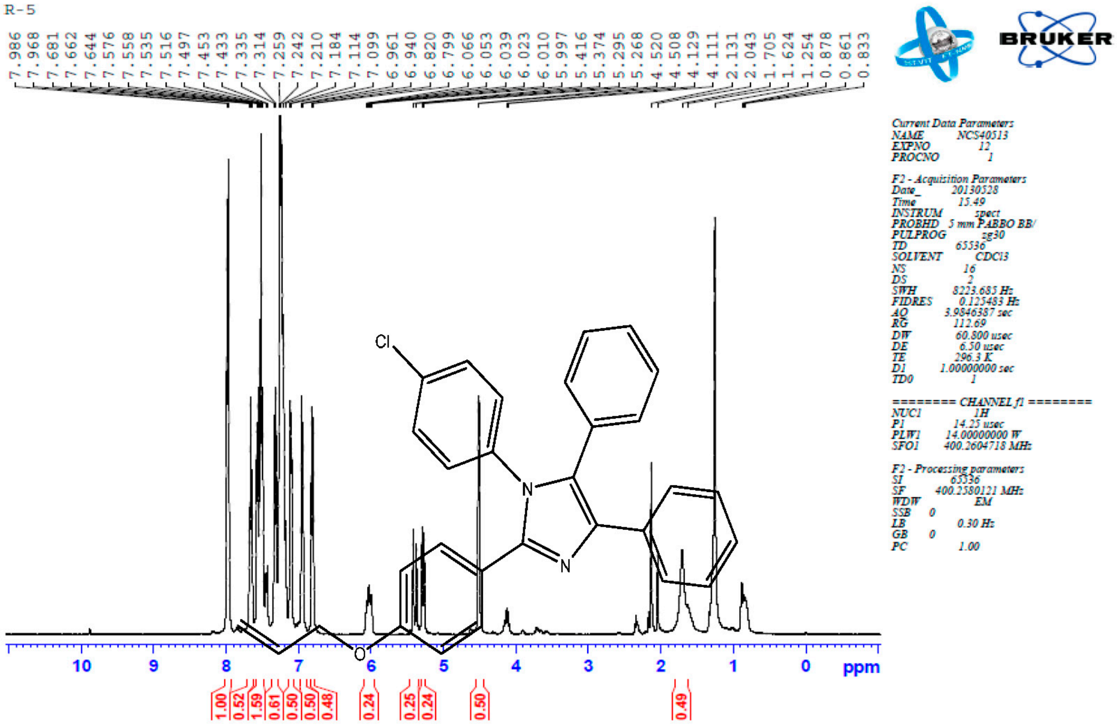

Figure S35. ^{1}H-NMR spectrum of 2-(4-(allyloxy)phenyl)-1-(4-chlorophenyl)-4,5-diphenyl-1*H*-imidazole (**16**).

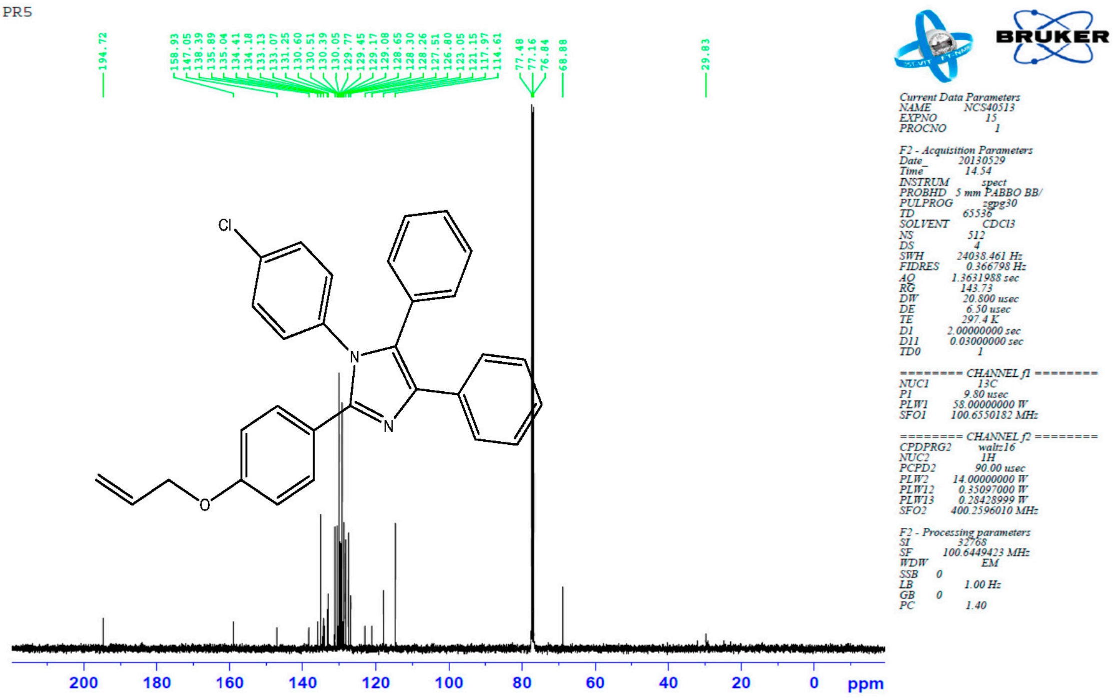

Figure S36. ^{13}C-NMR spectrum of 2-(4-(allyloxy)phenyl)-1-(4-chlorophenyl)-4,5-diphenyl-1*H*-imidazole (**16**).

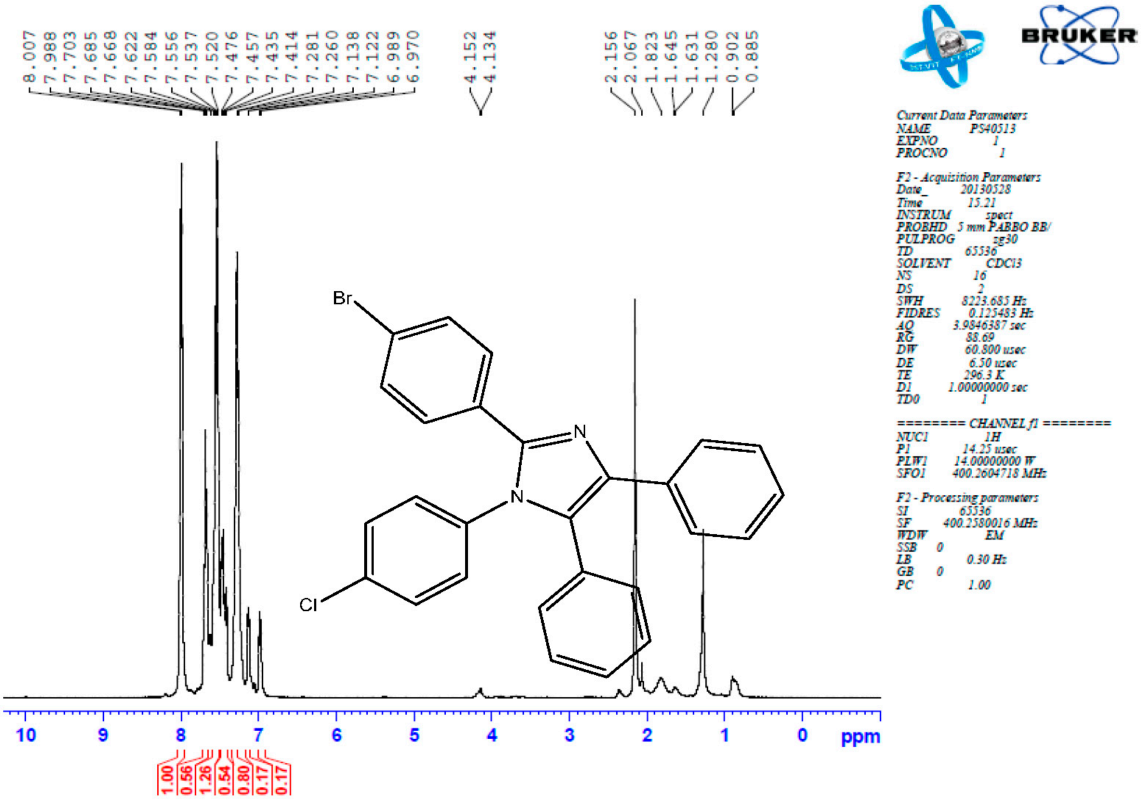

Figure S37. ^{1}H-NMR spectrum of 2-(4-bromophenyl)-1-(4-chlorophenyl)-4,5-diphenyl-1*H*-imidazole (**17**).

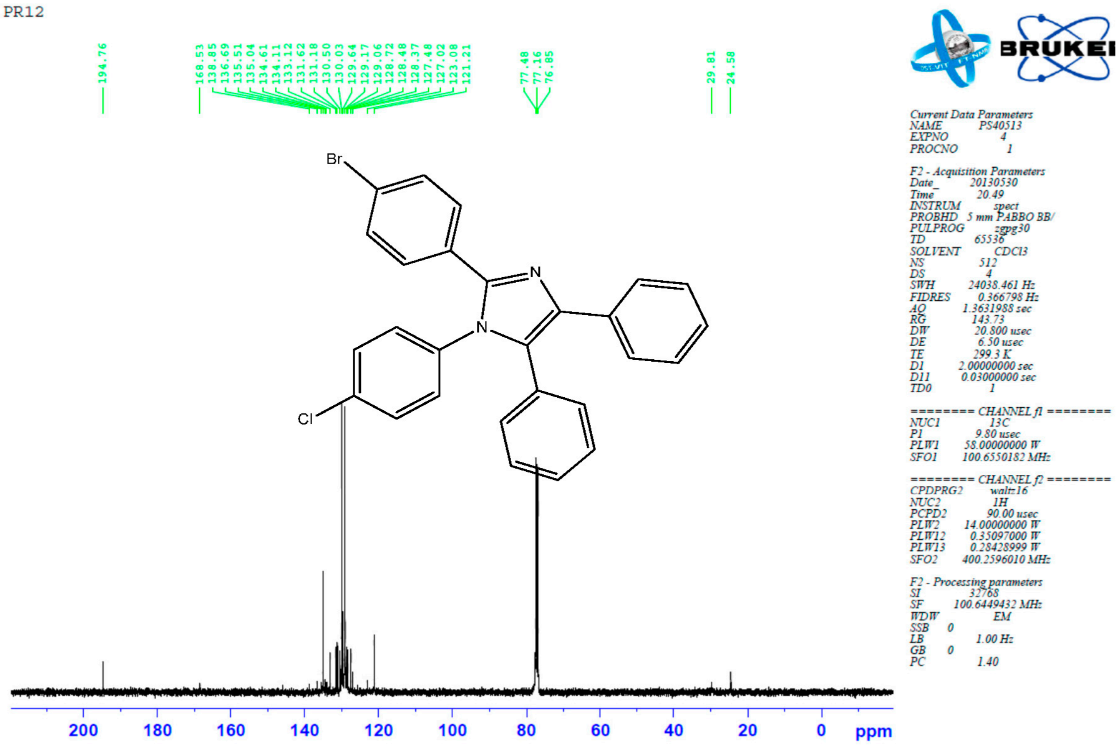

Figure S38. ^{13}C-NMR spectrum of 2-(4-bromophenyl)-1-(4-chlorophenyl)-4,5-diphenyl-1*H*-imidazole (**17**).

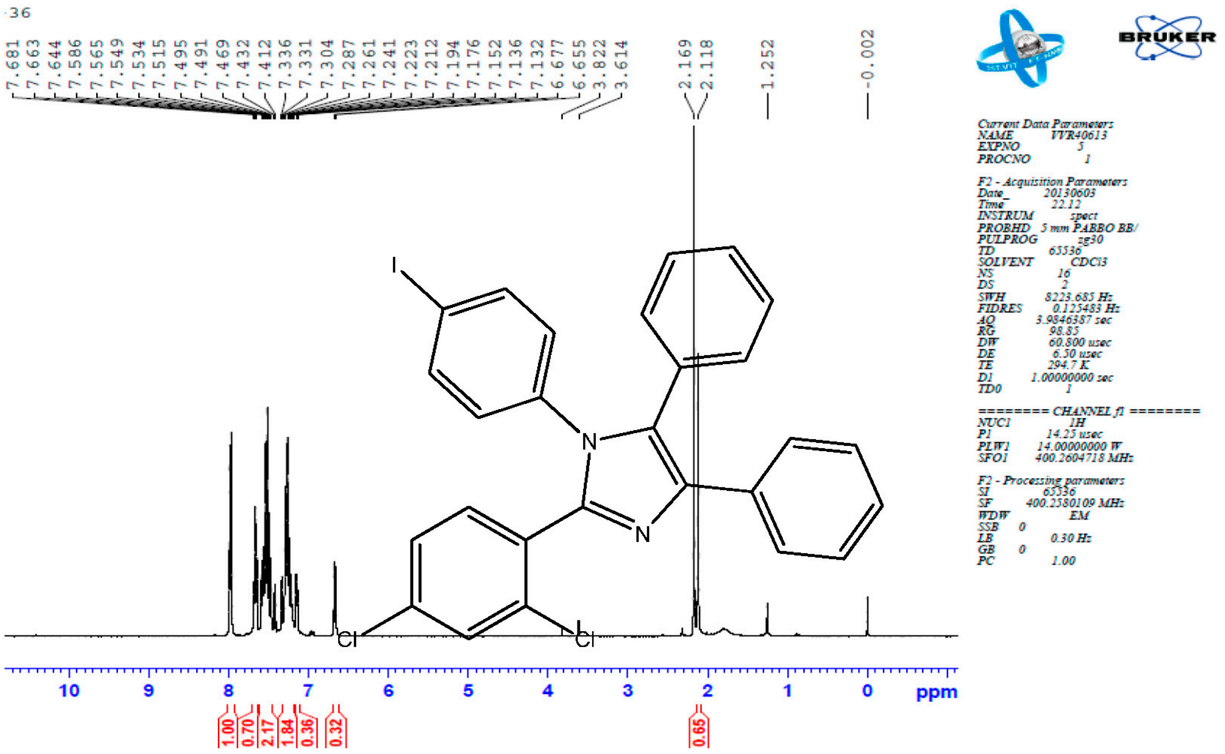

Figure S39. ^{1}H-NMR spectrum of 2-(2,4-dichlorophenyl)-1-(4-iodophenyl)-4,5-diphenyl-1*H*-imidazole (**18**).

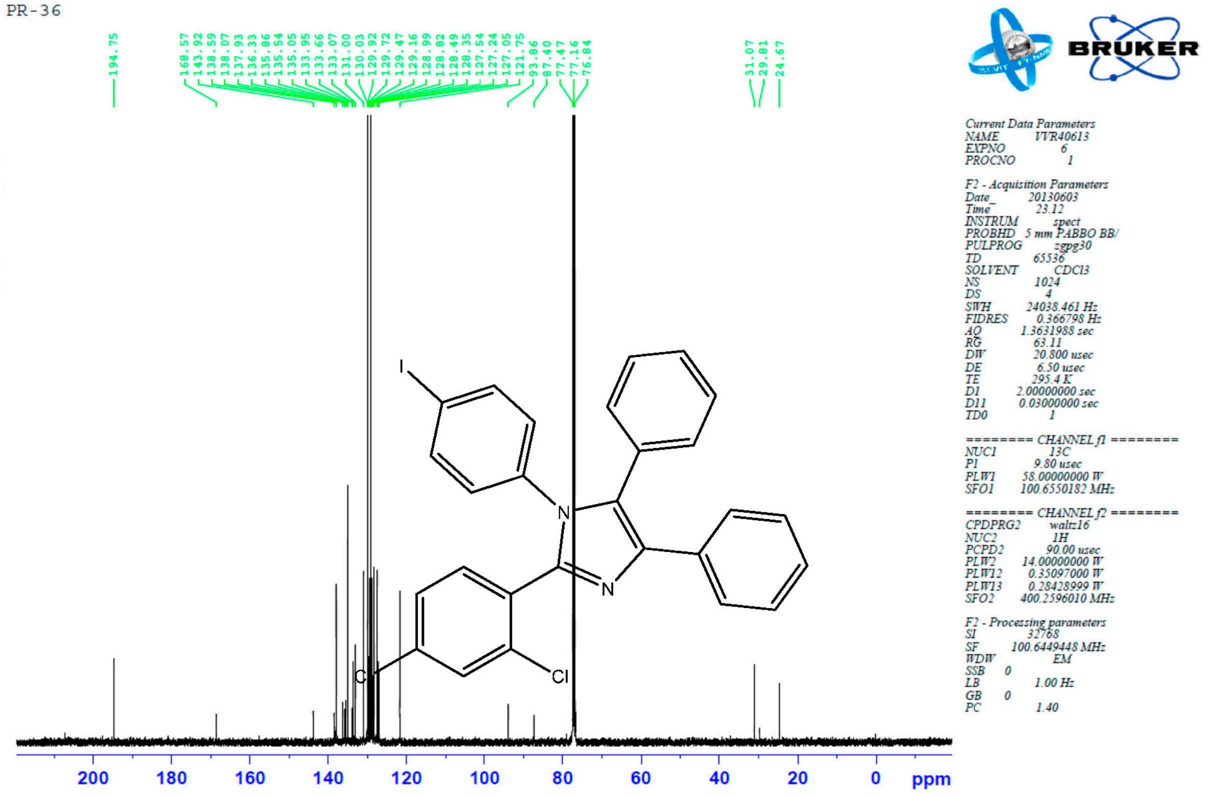

Figure S40. ^{13}C-NMR spectrum of 2-(2,4-dichlorophenyl)-1-(4-iodophenyl)-4,5-diphenyl-1*H*-imidazole (**18**).

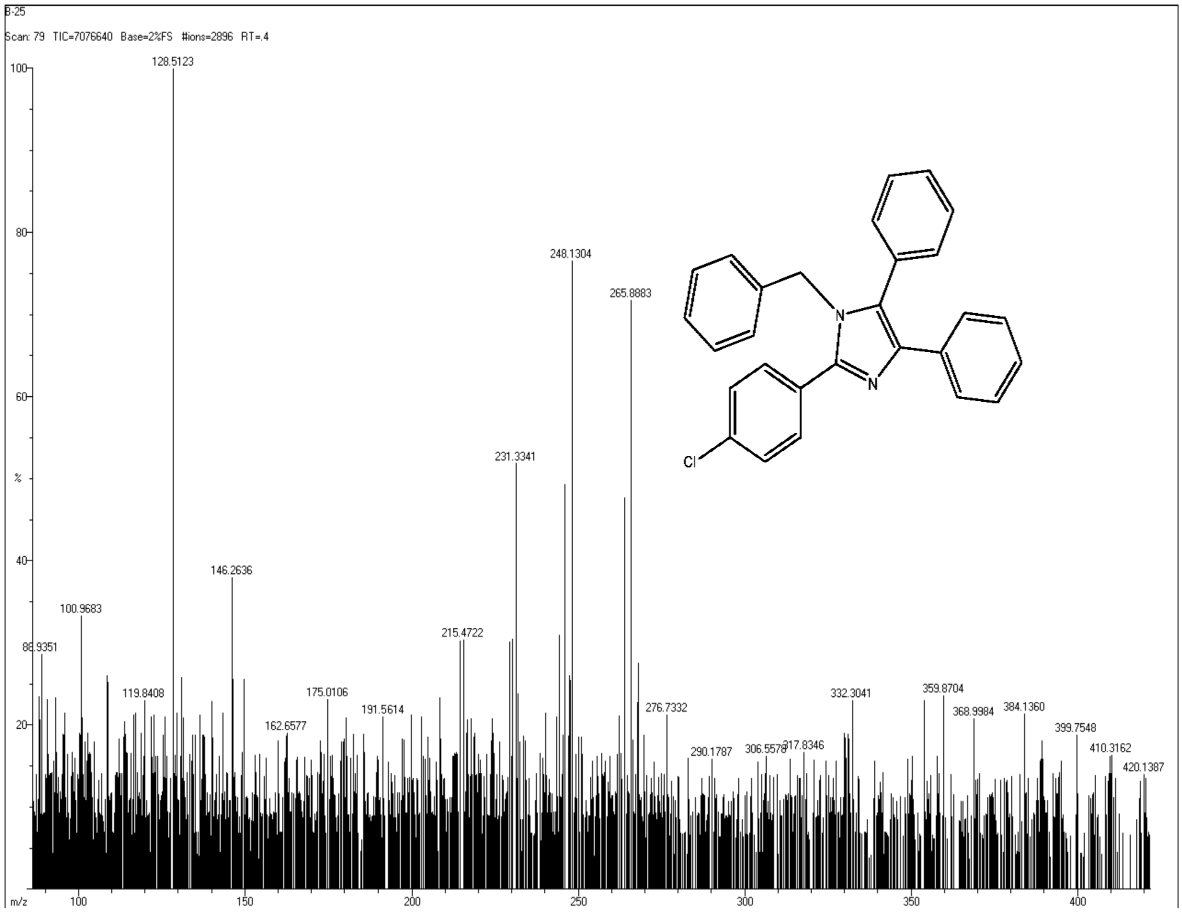

Figure S41. HRMS spectrum of 1-benzyl-2-(4-chlorophenyl)-4,5-diphenyl-1*H*-imidazole (**2**).

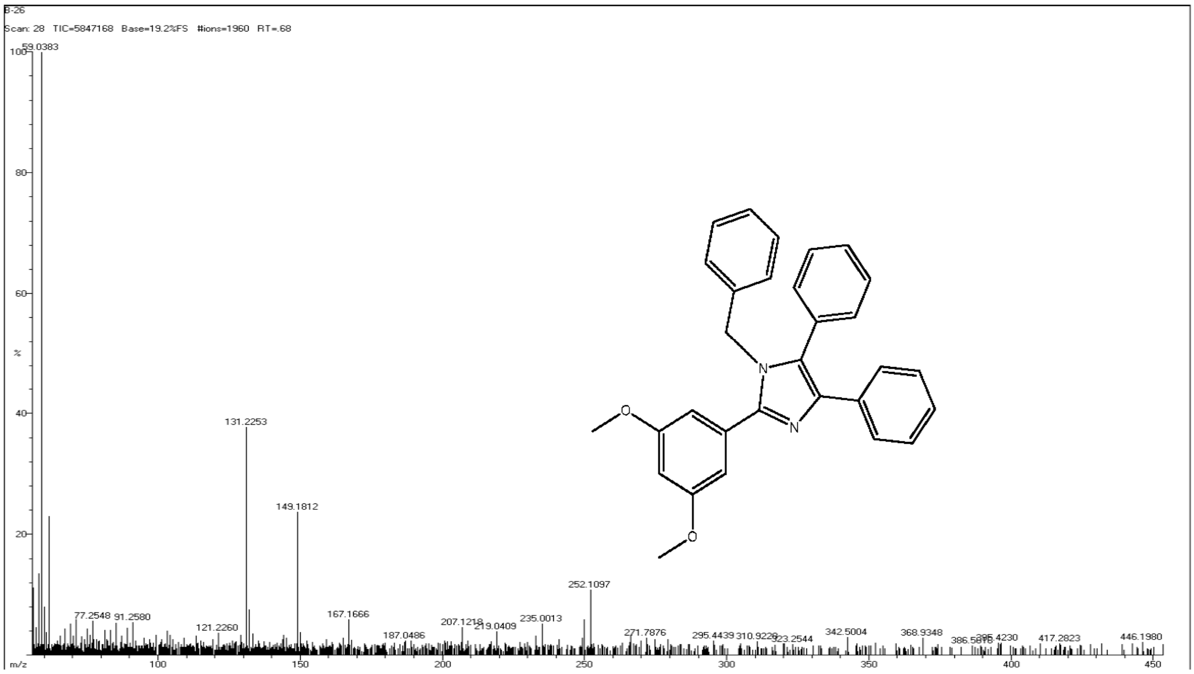

Figure S42. ^{1}H-NMR spectrum of 1-benzyl-2-(3,5-dimethoxyphenyl)-4,5-diphenyl-1*H*-imidazole (**4**).

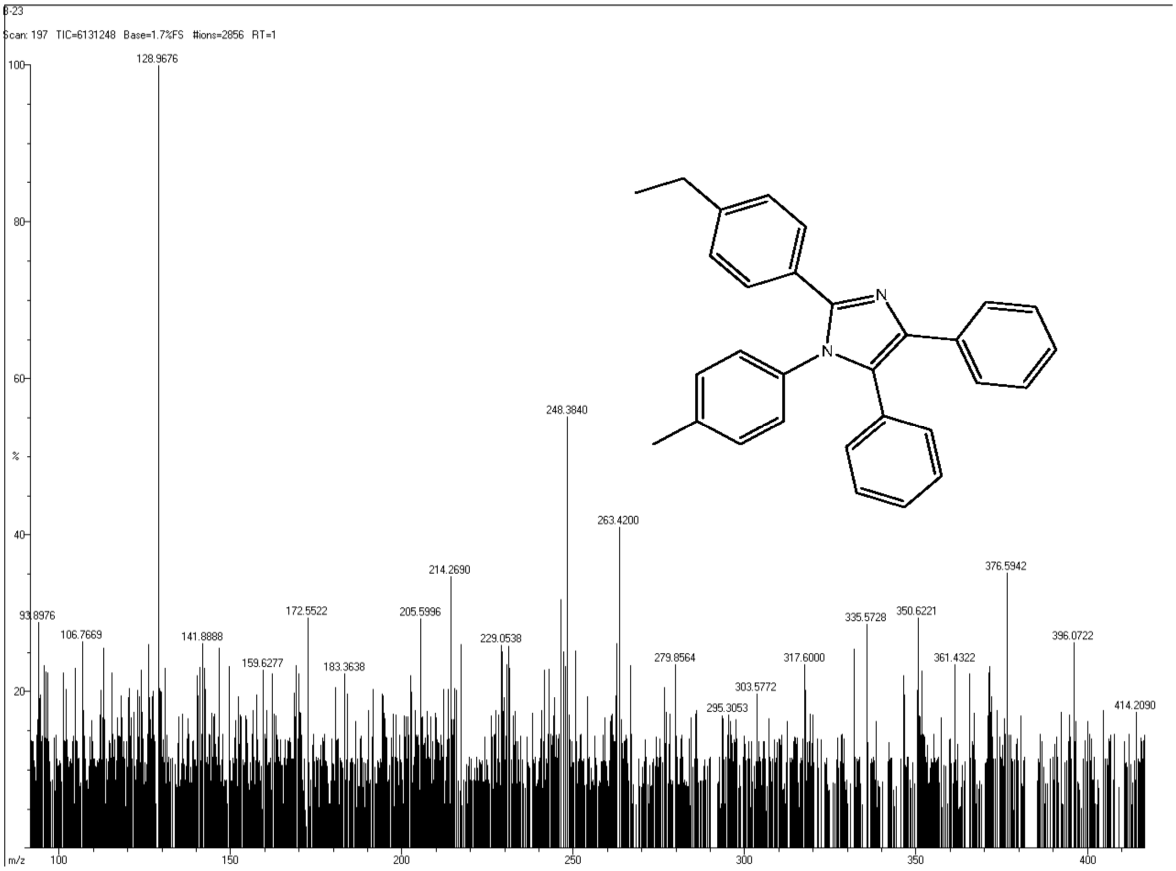

Figure S43. HRMS spectrum of 2-(4-ethylphenyl)-4,5-diphenyl-1-(*p*-tolyl)-1*H*-imidazole (**7**).

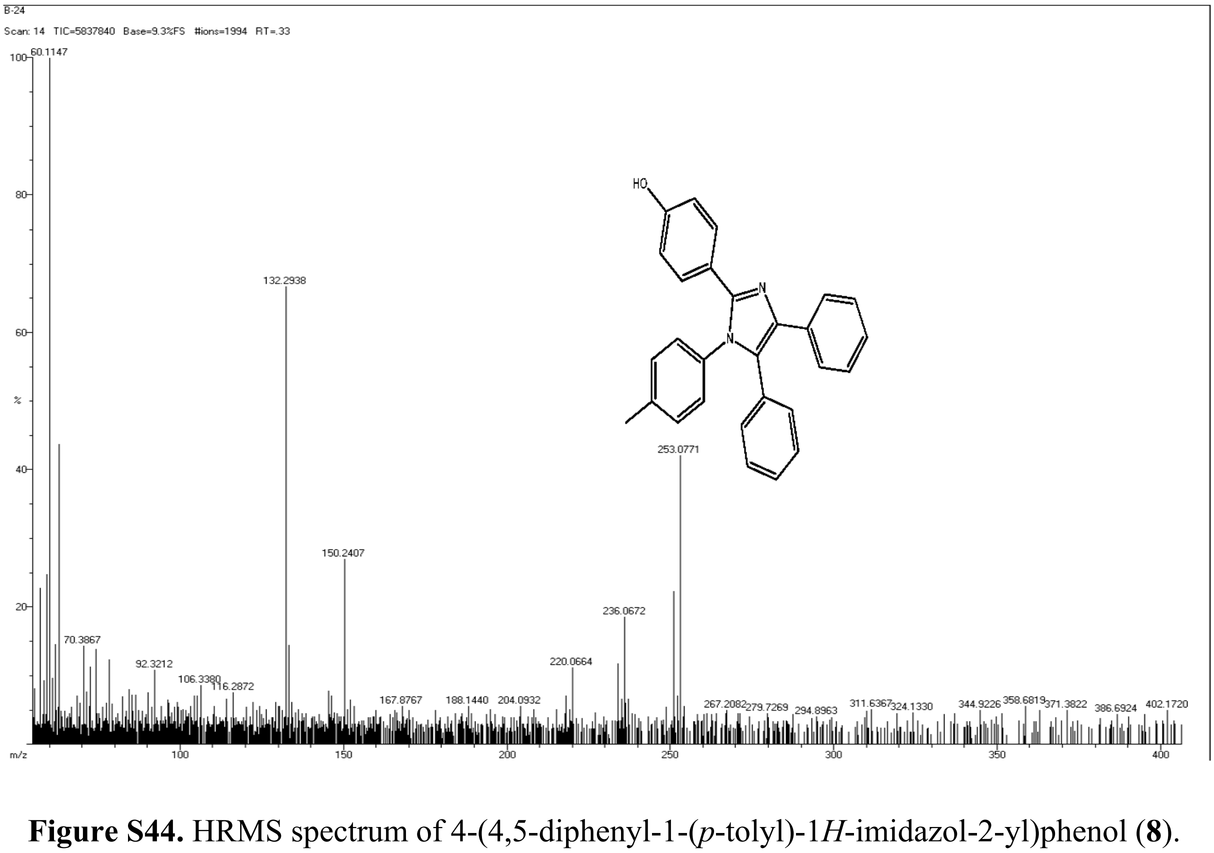

Figure S44. HRMS spectrum of 4-(4,5-diphenyl-1-(*p*-tolyl)-1*H*-imidazol-2-yl)phenol (**8**).

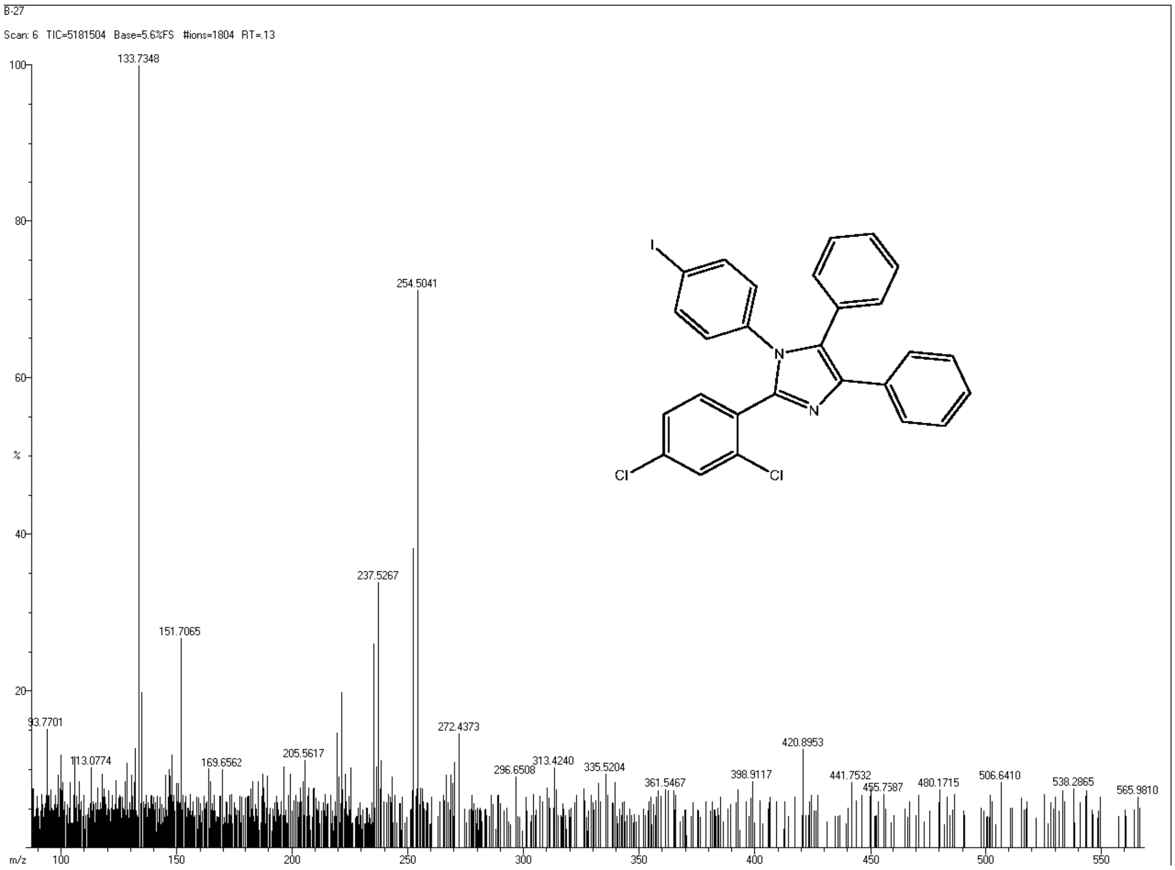

Figure S45. HRMS spectrum of 2-(2,4-dichlorophenyl)-1-(4-iodophenyl)-4,5-diphenyl-1*H*-imidazole (**18**).
